# Imputing causality and clonal dynamics from single-cell transcriptomics in paroxysmal nocturnal hemoglobinuria

**DOI:** 10.1038/s41375-026-02914-5

**Published:** 2026-04-22

**Authors:** Hiroki Mizumaki, Shouguo Gao, Zhijie Wu, Fernanda Gutierrez-Rodrigues, Lemlem Alemu, Diego Quinones Raffo, Ivana Darden, Olga Rios, Jennifer Lotter, Sachiko Kajigaya, Jibran Durrani, Emma M. Groarke, Bhavisha A. Patel, Neal S. Young

**Affiliations:** 1 Hematology Branch, National Heart, Lung, and Blood Institute, National Institutes of Health, Bethesda, MD USA; 2 Division of Hematology, Sylvester Comprehensive Cancer Center, University of Miami Miller School of Medicine, Miami, FL USA

**Keywords:** Translational research, Haematopoietic stem cells

## Abstract

Paroxysmal nocturnal hemoglobinuria (PNH) originates from hematopoietic stem cells (HSCs) harboring somatic mutations in the phosphatidylinositol glycan class A (*PIGA*) gene. Clonal expansion of *PIGA*-mutated cells occurs uniquely in the setting of bone marrow (BM) failure, but specific pathophysiologic mechanisms remain unclear. We performed single-cell RNA sequencing (scRNA-seq) of BM cells from patients with large (> 50%) and small (10–50%) PNH cell fractions. In patients with large PNH cell fractions, phenotypically normal hematopoietic stem and progenitor cells (HSPCs) upregulated immune response and apoptosis pathways and downregulated cell-cycling pathways compared with PNH-type HSPCs. BM effector cells upregulated immune response pathways, and cell-cell communication between effector cells and normal HSPCs was greater than in controls. In contrast, in patients with small PNH cell fractions, transcriptional changes in normal HSPCs were reversed: downregulation of immune response pathways and upregulation of the cell-cycling pathways. Notably, transcriptional differences associated with PNH cell fractions were primarily in normal HSCs, whereas PNH-type HSCs showed similar transcriptional profiles between patients with large and small PNH cell fractions. These results implicate immunological negative selection against normal HSCs in PNH. Error-corrected DNA sequencing of patients’ blood samples identified multiple *PIGA* mutations in each patient, consistent with strong selection for the resulting phenotype.

## Introduction

Paroxysmal nocturnal hemoglobinuria (PNH) is a rare life-threatening hematologic disease, characterized by a triad of clinical manifestations: intravascular hemolysis, bone marrow (BM) failure, and venous thrombosis [1, 2]. PNH is the paradigm of an acquired genetic disease [3]; PNH arises in hematopoietic stem cells (HSCs) from an acquired somatic mutation of the phosphatidylinositol glycan class A (*PIGA*) gene. PNH occurs almost exclusively on the background of immune BM failure. *PIGA* mutations result in loss of the glycosylphosphatidylinositol anchored proteins (GPI-APs) on the cell surface of affected cells. *PIGA*-mutated (mt*PIGA*) HSCs clonally expand and can represent the majority of peripheral blood (PB) mature cells in PNH patients, but most GPI-AP deficient (GPI(−)) PNH clones are small and stable over time [4, 5]. In animal models, mt*PIGA* HSCs are capable of multilineage hematopoietic reconstitution but fail to undergo clonal dominance over time, indicating mt*PIGA* HSCs have no cell-intrinsic growth advantage [6–8]. These findings strongly suggest that the *PIGA* mutations alone are necessary but not sufficient to develop the disease PNH, and expansion of mt*PIGA* HSCs is dependent on selection within a specific BM environment. In our previous, early study utilizing gene chips and CD34^+^ cells from classical, mainly hemolytic PNH patients, GPI-AP positive (GPI( +)) CD34^+^ cells showed upregulation in genes involved in apoptosis compared to GPI(−) CD34^+^ cells, indicating relatively increased survival of GPI(−) PNH-type CD34^+^ cells compared to GPI(+) normal CD34^+^ cells in PNH [9].

Single-cell RNA sequencing (scRNA-seq) is a powerful method to deconstruct a disease like PNH: scRNA-seq requires minimal sample manipulation, avoids ex vivo cell culture artifacts, and allows for detection of rare cell types even in sparse BM samples [10]. scRNA-seq and other single cell methods are largely free of a priori bias as utilizing open-ended approaches to data accrual and analysis. We performed scRNA-seq of enriched hematopoietic stem and progenitor cells (HSPCs) and BM immune cells from PNH patients to understand the disease pathophysiology at high multi-dimensional resolution. In addition, we assessed the dynamics of mt*PIGA* clones within patients over time by DNA sequencing.

## Methods

For full description of experimental procedures and analytical methods, see Supplementary Methods.

### Subjects and samples

Nine fresh BM samples were obtained from eight PNH patients with > 10% of GPI(−) granulocytes (2 males and 6 females; median age, 43 [range, 21-67]), and five age- and sex-matched healthy donors (2 males and 3 females; median age, 47 [range, 29-61]) (Table 1). Flow cytometric sorting of GPI(−) and GPI(+) cells from subjects was performed immediately following isolation of BM mononuclear cells (BMMNCs). Our sorting strategy is illustrated in Supplementary Fig. 1. Sorted cells were processed through the Chromium Controller (10x Genomics, Pleasanton, CA, USA). scRNA-seq libraries from the subjects were constructed using the Chromium Single Cell 3’ Reagent Kits (10x Genomics), according to the manufacturer’s instructions. The constructed libraries were sequenced with the NovaSeq 6000 system (Illumina, San Diego, CA, USA).

**Table 1:** Clinical and laboratory characteristics of PNH patients at the time of BM examination.

ID	Age (years) /Sex	Diagnosis	Prior IST	Current IST	Time from diagnosis (months)	AA status	Complement inhibitors	RBC transfusion dependance	GPI-AP(−) granulocytes (%)	GPI-AP(−) monocytes (%)	GPI-AP(−) RBC (%)	WBC (4.63 - 6.08 ×10^3^/µL)	ANC (1.78 - 5.38 ×10^3^/µL)	Hb (13.7 - 17.5 g/dL)	Reti (26 - 95 ×10^3^/µL)	Platelets (161 - 347 ×10^3^/µL)	LDH (125 - 220 U/L)
PNH 3	43/M	AA/PNH	hATG, CsA, EPAG	none	84	CR	none	no	15.99	13.53	type II 0.09, type Ⅲ 5.48	4.54	2.54	14.7	72.8	234	203
PNH 4	60/F	AA/PNH	hATG, CsA	none	83	CR	Ravulizmab, Danicopan	no	93.76	93.44	type II 1.70, type III 88.94	3.21	1.57	12.2	94.1	147	214
PNH 5	67/F	AA/PNH	rATG, CsA, Alemtuzumab	none	175	CR	none	no	22.69	15.51	type II 0.16, type III 4.35	4.47	2.16	13	66.1	217	329
PNH 6	36/F	AA/PNH	hATG, CsA, EPAG	CsA	6, 12	PR	none	no	30.54	24.84	type II 0.57, type III 7.88	5.88	4.86	10	102.3	61	561
PNH 7	21/F	AA/PNH	hATG, CsA, EPAG	none	52	CR	none	no	99.15	93.5	type II 3.78, type III 90.69	5.14	2.84	14.3	47.2	132	234
PNH 8	58/F	AA/PNH	CsA	CsA	20	PR	Ravulizumab	yes	41.48	60.66	type II 1.40, type III 13.71	3.99	2.63	7.8	141.3	93	312
PNH 9	29/F	Classical PNH	none	none	N/A	N/A	Ravulizumab	no	91.0	N/A	N/A	4.21	2.44	8.8	191.2	74	259
PNH 10	67/M	AA/PNH	hATG, CsA, EPAG	CsA	129	PR	Ravulizumab	no	45.62	72.06	type II 0.36, type III 6.06	2.25	2.14	9.3	48.1	84	236

*AA* aplastic anemia, *ANC* absolute neutrophil count, *BM* bone marrow, *CsA* cyclosporine, *CR* complete response, *EPAG* eltrombopag, *F* *female*, *GPI-AP* glycosylphosphatidylinositol anchored protein, *hATG* horse anti-thymogloblin, *Hb* hemoglobin, *IST* immunosuppressive therapy, *LDH* Lactate Dehydrogenase, *M* male, *N/A* not available, *rATG* rabbit anti-thymogloblin, *RBC* red blood cells, *Reti* reticulocytes, *PNH* paroxysmal nocturnal hemoglobinuria, *PR* partial response, *WBC* white blood cells.

Written informed consent was obtained from all patients under protocols approved by the Institutional Review Boards of the National Heart, Lung, and Blood institute (NHLBI) (www.clinicaltrials.gov; NCT05012111, NCT01623167, and NCT04304820), in accordance with the Declaration of Helsinki. Healthy donors were enrolled as controls under NHLBI protocol NCT00442195.

### scRNA-seq data analysis

#### Data preprocessing

Alignment, barcode assignment, and unique molecular identifier counting were performed using the Cellranger pipeline (http://software.10xgenomics.com/single-cell/overview/welcome). Two samples (PNH 6-2 GPI(−) CD34^+^ cells and PNH 7 GPI( + ) CD34^+^ cells) that failed QC threshold due to low recovered cell numbers ( < 200 cells) were excluded from further downstream analyses. Sequencing quality metrics are provided in Supplementary Table 1.

#### Downstream analyses

Dimensionality reduction and clustering were performed by principal component analysis and visualized with uniform manifold approximation and projection (UMAP) in the Seurat package (http://satijalab.org/seurat/, v4.0.4) [11]. Cell type identity was assigned to each cluster based on significance of overlap between signature genes of BMMNCs [12], HSPCs [13], and cluster-specific genes (Fisher’s exact test). Monocle [14] was used to reconstruct a differentiation continuum of cells and to order individual cells’ differentiation for pseudotime analysis. Differences of cell abundances between samples of patients and healthy donors were analyzed by the differential abundance testing with miloR [15]. An expression variance for each gene was partitioned using variancePartition [16]. A multidimensional scaling plot (MDS) was generated by the function of the plotMDS in edgeR package [17]. fgsea [18] was used for Gene Set Enrichment Analysis (GSEA) to interpret gene set enrichment and pathways of defined differentially expressed genes. Cell-cell interactions based on expression of known ligand-receptor pairs in different cell types were calculated using CellPhoneDB v5 [19].

### DNA sequencing

Patients were screened for somatic mutations in PB (seven granulocyte and one PB mononuclear cells samples) using an error-corrected sequencing (ECS) panel comprised of genes associated with aplastic anemia (AA) and myeloid malignancies, as previously described (VariantPlex ArcherDx; Integrated DNA Technologies, Newark, NJ, USA) (Supplementary Table 2) [20]. Libraries were sequenced on the NovaSeq 6000 system (Illumina; average coverage of 600× deduplicated reads), and variants with a deduplication ratio > 3:1 and variant allele frequency (VAF) ≥ 0.5% were included in the analysis. De novo variants (VAF ≥ 0.5%) identified at any timepoint were tracked in available serial samples and retrospectively included in the analysis if detectable at VAF ≥ 0.1%.

### Flow cytometry profiling of BMMNCs

To identify PNH-type cell populations in each cell subtype, BMMNCs from eight PNH patients were stained with antibodies (Supplementary Methods), and acquired on the BD LSRFortessa (BD Biosciences, Franklin Lakes, NJ, USA). Data were analyzed using FlowJo software (FlowJo version 10.2; Ashland, OR, USA). Patients were classified into large or small PNH cell fraction groups based on proportions of GPI(−) HSPCs in the BM at the time of sampling for scRNA-seq. Those with a > 50% of GPI(−) HSPCs were defined as large fractions, a clear cutoff to separate the cohort into two groups (Supplementary Fig. 2A, 2B) and a threshold that has been used in clinical contexts in PB granulocytes [21, 22].

### Statistics

Statistical analyses were performed as described in the figure legends. Comparison between groups was performed using the GraphPad Prism (v10.2.0; GraphPad software, La Jolla, CA, USA), and results were shown as mean ± standard derivation (SD).

## Results

### Patients

**Fig. 1:**
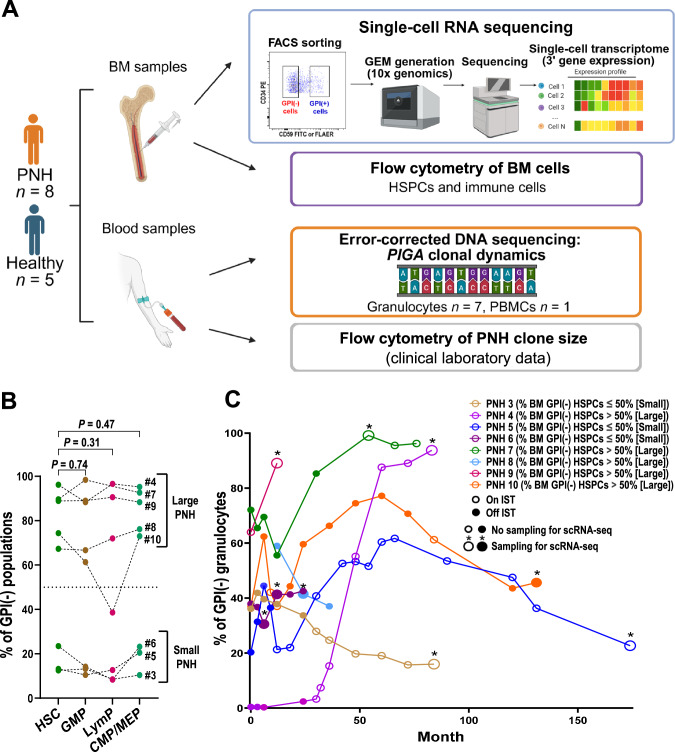
Single-cell RNA sequencing (scRNA-seq), flow cytometry profiling, and error-corrected DNA sequencing (ECS) of samples from PNH patients and healthy donors. **A** Overview of the experimental workflow. scRNA-seq and flow cytometric profiling were performed on bone marrow (BM)-derived cells. *PIGA* clonal dynamics were evaluated by bulk targeted DNA sequencing of peripheral blood (PB) cells. Percentages of GPI(−) granulocytes in PB were obtained from clinical laboratory data. This figure was created with BioRender.com. **B** A percentage of each GPI(−) hematopoietic stem and progenitor cell (HSPC) type relative to the total number of Lin^-^CD34^+^ cells derived from PNH patients (*n* = 8). Patients were stratified into two groups based on  a proportion of GPI(−) HSPCs in the BM assessed by flow cytometry at the time of sampling for scRNA-seq: patients with large PNH cell fractions ( > 50% of GPI(−) HSPCs) and patients with small PNH cell fractions (10–50% of GPI(−) HSPCs). *P* values were calculated using the Wilcoxon matched-pairs signed-rank test. **C** Longitudinal changes in the percentage of GPI(−) granulocytes in PB of PNH patients. Large symbols with asterisks indicate the time of sample collection for scRNA-seq. Small symbols indicate the time of sample collection for clinical flow cytometry of GPI(−) granulocytes. Closed and open symbols indicate on- and off-immunosuppressants, respectively. BM bone marrow, CMP common myeloid progenitor, FACS fluorescence-activated cell sorting, GEM Gel Bead-in-Emulsion, GMP granulocyte-monocytic progenitor, GPI glycosylphosphatidylinositol, HSC hematopoietic stem cell and multipotent progenitor, IST immunosuppressive therapy, LymP lymphocyte progenitor, MEP megakaryocyte-erythrocyte progenitor, MLP multi-lymphoid progenitor, PBMCs peripheral blood mononuclear cells.

Eight patients were included for scRNA-seq and flow cytometry analysis of BM samples (Fig.1A and Table 1). Five patients had large PNH cell fractions ( > 50% of GPI(−) HSPCs) and three had small PNH cell fractions (10**-**50% of GPI(−) HSPCs) (Fig. 1B). All but one patient had a history of AA, and AA/PNH patients had been treated with immunosupressive therapy (IST) at median 82.6 months (range, 6.1–175.5 months) prior to sample collection for scRNA-seq. At the time of sample collection, AA was in remission without ongoing treatment in five patients. Of the five patients with large PNH cell fractions, four patients were receiving treatment with anti-complement therapy with control of hemolysis in all the patients. Clonal dynamics of GPI(−) granulocytes by clinical flow cytometry were variable among patients; there was no consistent effect of IST on size of GPI(−) granulocytes during long-term follow-up (Fig. 1C).

### scRNA-seq of HSPCs in PNH patients

**Fig. 2:**
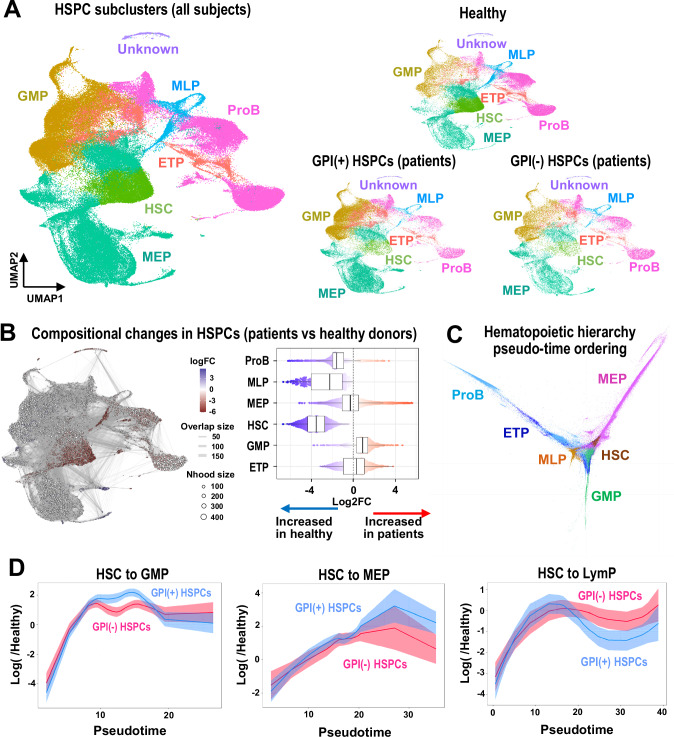
scRNA-seq analysis of HSPCs from PNH patients and healthy donors. **A** Uniform Manifold Approximation and Projection (UMAP) plots of 138,015 HSPCs from all subjects (*n* = 13, left), 56,545 HSPCs derived from healthy donors (*n* = 5, upper right), and 51,742 GPI(+) (lower middle) and 29,728 GPI(−) HSPCs (lower right) derived from PNH patients (*n* = 8). Leiden clusters based on gene expression are shown and colored by HSPC subtypes. **B** Differential abundance analysis of HSPCs using Milo. The neighborhood graph (left) shows neighborhoods (Nhoods) within the HSPC populations, with node colors indicating log2-fold change (log2FC) in abundance between PNH patients and healthy donors. Significant changes are colored in blue and red. Nondifferential abundance Nhoods (a false discovery rate [FDR] ≥ 0.10) are shown in white. A Beeswarm and box plot (right) shows the distribution of log2FC differences in neighborhoods in different cell type clusters. Colors are represented similarly to the neighborhood graph. A box plot shows median and interquartile ranges (IQR), with whiskers extending to most extreme values within 1.5*IQR. **C** Reconstitution of the hematopoietic hierarchy based on pseudotime ordering. Cell types are colored by HSPC subtypes (HSC, MLP, MEP, GMP, ETP, and ProB). **D** Dynamic changes in ratios of GPI(−) and GPI(+) HSPCs compared with healthy donor HSPCs along differentiation. The x axis represents pseudotime ordering from HSCs to lineage-restricted progenitors, and the y axis represents ratios of cell numbers of GPI(−) or GPI(+) HSPCs to those of healthy donor HSPCs on a log scale. ETP early T lineage progenitor, GMP granulocyte-monocytic progenitor, HSC hematopoietic stem cell and multipotent progenitor, ProB B lymphocyte progenitor, MEP megakaryocyte-erythrocyte progenitor, MLP multi-lymphoid progenitor.

We examined transcriptomes of enriched lineage^−^CD34^+^ HSPCs from eight PNH patients and five healthy donors by scRNA-seq. After quality control, a total of 138,015 cells were retained for further analyses, comprising 29,728 GPI(−) and 51,742 GPI(+) cells from patients, and 56,545 cells from healthy donors (Fig. 2A). From published cell type signatures [13], we deconvoluted single cells in a heterogenous HSPC subsets as HSCs, megakaryocyte-erythrocyte progenitors (MEPs), granulocyte-monocytic progenitors (GMPs), B lymphocyte progenitors (ProBs), early T lineage progenitors (ETPs), and multi-lymphoid progenitors (MLPs) (Fig. 2A and Supplementary Fig. 3A).

Differential abundance analysis showed that PNH patients, most of whom also had a history of AA, had prominently decreased HSCs, MLPs, and ProBs compared to healthy donors (Fig. 2B). There was no difference in cell compositions between patients with large and small PNH cell fractions (Supplementary Fig. 3B). Flow cytometry confirmed a decrease of HSCs in PNH patients (Supplementary Fig. 4A). When the hematopoietic hierarchy was reconstructed by pseudo-temporal ordering, we observed anticipated three major differentiation trajectories: from HSCs to MEPs, to GMPs, and to lymphoid progenitors (Fig. 2C). In PNH patients, similar ratios of GPI(−) and GPI(+) HSPCs were observed along pseudotime trajectories in patients across all lineages, indicating similar differentiation potential (Fig. 2D); flow cytometry also showed similar percentages of GPI(−) cell populations across all HSPC subtypes in individual patients (Fig. 1B and Supplementary Fig. 4B).

Gene expression profiles of HSPCs from PNH patients and healthy donors were different by scRNA-seq analysis (Supplementary Fig. 5A). Differences were also apparent between GPI(−) and GPI(+) HSPCs within each patient, and notably between HSPCs from patients with small and large PNH cell fractions. A multidimensional scaling plot of whole-transcriptome profiles of HSPCs showed that healthy donors clustered tightly, indicating low inter-individual variability (Supplementary Fig. 5B). Paired GPI(−) and GPI(+) HSPCs from each PNH patient also grouped closely together. In contrast, HSPCs from different PNH patients—both GPI(−) and GPI(+)—were more widely dispersed, reflecting greater inter-patient heterogeneity, with GPI(+) HSPCs from patients with large versus small PNH cell fractions, showing a larger deviation from healthy donors than GPI(−) HSPCs (Supplementary Fig. 5C).

### Gene expression profiling of GPI(+) and GPI(−) HSPCs in patients with large PNH cell fractions

**Fig. 3:**
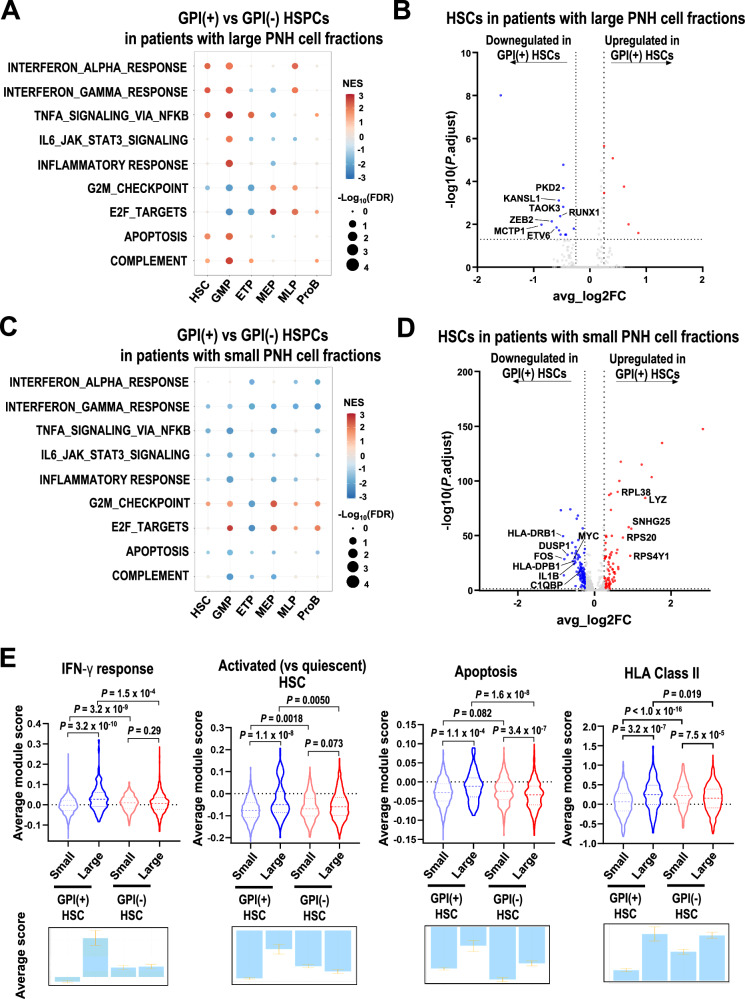
Differential gene expression between GPI(+) and GPI(−) HSPC subtypes in PNH patients. **A** Dot plot showing gene set enrichment scores across HSPC subtypes comparing GPI(+) and GPI(−) HSPCs in patients with large PNH cell fractions by Gene Set Enrichment Analysis (GSEA). A color scale indicates mean normalized enrichment score (NES) differences between two groups, and dot sizes indicate false discovery rate (FDR) values. Non-significant pathways (FDR ≥ 0.20) are shown in grey. **B** A volcano plot of differentially expressed genes between GPI(+) and GPI(−) HSCs in patients with large PNH cell fractions. A horizontal dotted line and vertical dotted lines represent an adjusted *P*-value (*p*adj) = 0.05 and absolute log2FC = 0.25, respectively. Genes upregulated and downregulated in GPI(+) HSCs compared to GPI(−) HSCs are highlighted in red and blue, respectively. **C** A dot plot of gene set enrichment scores across HSPC subtypes between GPI(+) and GPI(−) HSPCs in patients with small PNH cell fractions. **D** A volcano plot of differentially expressed genes between GPI(+) and GPI(−) HSCs in patients with small PNH cell fractions, generated as in (**B**). **E** Gene set module scores for the IFN-γ response pathway, activated HSC signature, the apoptosis pathway, and HLA class II genes in HSCs. Scores were shown for GPI(+) and GPI(−) HSCs in patients with small and large PNH cell fractions as violin plots, with mean module scores shown in bar charts at the bottom. *P* values were calculated with the two-sided unpaired Mann–Whitney *U* test. ETP, early T lineage progenitor, GMP granulocyte-monocytic progenitor, HSC hematopoietic stem cell and multipotent progenitor, ProB B lymphocyte progenitor, MEP megakaryocyte-erythrocyte progenitor, MLP multi-lymphoid progenitor.

GSEA analysis of differential genes between GPI(+) vs GPI(−) cells within HSPC subsets showed upregulation of the immune response pathways and downregulation of the cell-cycling pathways (G2M checkpoint and E2F targets) in GPI(+) cells from HSC and GMP subsets (Fig. 3A and Supplementary Table 3), consistent with our previous study [9]. Heterogeneous patterns of transcriptional changes in others HSPC subsets were observed. There were very few differentially expressed genes between GPI(+) and GPI(−) HSCs in patients with large PNH cell fractions: genes essential for maintaining HSC functions, such as *ETV6* and *ZEB2*, were downregulated in GPI(+) HSCs, as compared to GPI(−) HSCs (Fig. 3B).

### Gene expression profiling of GPI(+) and GPI(−) HSPCs in patients with small PNH cell fractions

In PNH patients with small PNH cell fractions, GSEA analysis showed downregulation of genes involved in immune response pathways and upregulation of genes involved in cell-cycling pathways in GPI(+) cells across HSPC subtypes, as compared to GPI(−) cells in corresponding HSPC subtypes, a trend opposite to GPI(+) HSPCs from patients with large PNH cell fractions (Fig. 3C and Supplementary Table 4).

Genes associated with immune responses, such as *IL1B* and *FOS*, were downregulated in GPI(+) HSCs as compared to GPI(−) HSCs in patients with small PNH cell fractions, consistent with GSEA data (Fig. 3D). Notably, HLA class II genes, such as *HLA-DRB1* and *HLA-DPB1*, were also downregulated in GPI(+) HSCs, which was not observed in GPI(+) HSCs from patients with large PNH cell fractions.

### Transcriptional differences in HSCs between patients with small and large PNH cell fractions

Transcriptional profiles of HSCs in patients with large PNH cell fractions differed markedly from those in patients with small PNH cell fractions. However, it remained unclear whether transcriptional changes in either or both GPI(+) and GPI(−) HSCs varied according to PNH clone sizes. To directly compare transcriptional features across GPI(+) and GPI(−) HSCs of patients with small and large PNH cell fractions, gene module scores were calculated for IFN-γ response, activated HSC gene signatures, cell apoptosis, cell-cycling, HLA class II genes, and cellular stress responses (unfolded protein responses). GPI(+) HSCs in patients with large PNH cell fractions had the highest IFN-γ response gene module scores among all GPI(+) and GPI(−) HSC populations, and these scores were significantly higher than those of GPI(+) HSCs from patients with small PNH cell fractions (Fig. 3E). In contrast, IFN-γ response scores were equivalent between GPI(−) HSCs from patients with large and small PNH cell fractions. Similar trends were observed for gene module scores associated with cell apoptosis, activated HSC gene signature, cell cycling, and cellular stress responses (Fig. 3E and Supplementary Fig. 5D). In addition, HLA class II gene scores of GPI(+) HSCs from patients with small PNH cell fractions were significantly lower than those of GPI(+) HSCs from patients with large PNH cell fractions, whereas score differences between GPI(−) HSCs from patients with large and small PNH cell fractions were minimal (Fig. 3E). In summary, transcriptional changes in GPI(+)—wild type-HSCs, not in GPI(−)—*PIGA* mutated-HSCs, resulted in transcriptional differences between GPI(+) and GPI(−) HSCs in PNH.

### scRNA-seq of BM immune cells

**Fig. 4:**
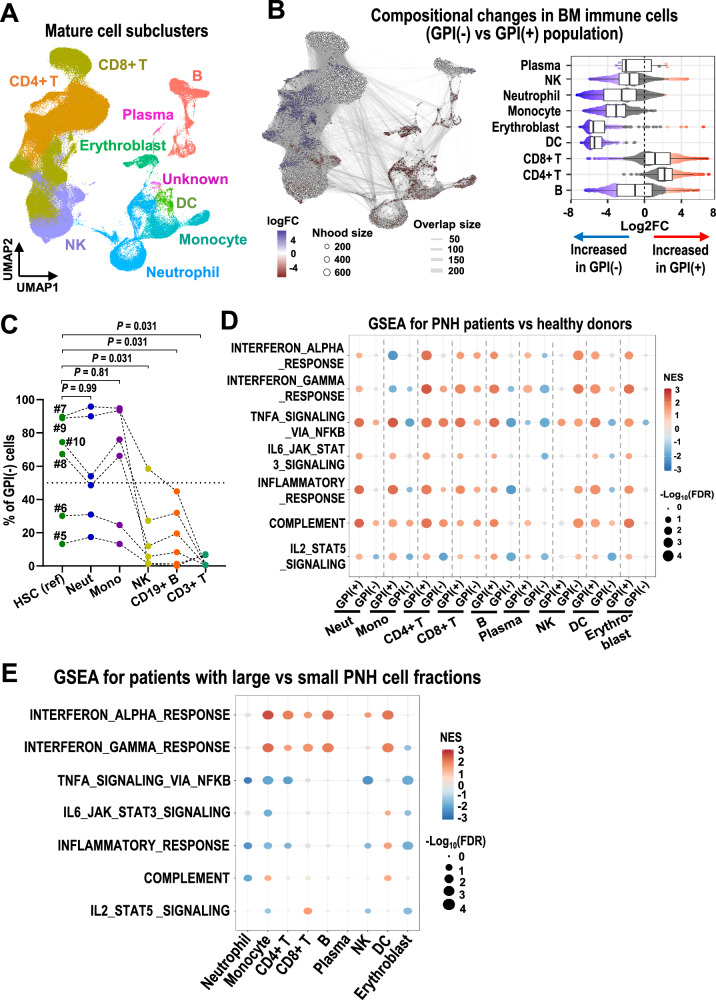
scRNA-seq of BM immune cells from PNH patients and healthy donors. **A** A Uniform Manifold Approximation and Projection (UMAP) plot of 190,032 BM immune cells from all subjects. Leiden clusters based on 3’ gene expression are shown, colored by major cell types. **B** Differential abundance analysis of BM immune cells using Milo. The neighborhood graph (left) shows neighborhoods (Nhoods) within BM immune cells, with node colors indicating log2FC between GPI(+) and GPI(−) cell populations in PNH. Significant changes are colored in blue and red. Nondifferential abundance Nhoods (false discovery rate [FDR] ≥ 0.10) are shown in white. A Beeswarm and box plot (right) shows the distribution of log2FC differences in different cell type clusters as in Fig. 2B. **C** Percentages of GPI(−) cell populations relative to the total number of cells in each immune cell type derived from PNH patients (*n* = 6). *P*-values were calculated using the Wilcoxon matched-pairs signed rank test. **D** A dot plot showing gene set enrichment scores across BM immune cell subtypes in PNH patients by GSEA. A color scale indicates mean normalized enrichment score (NES) differences between PNH patients and healthy donors, and dot sizes indicate FDR values. Non-significant pathways (FDR ≥ 0.20) are shown in grey. **E** A dot plot showing gene set enrichment scores across BM immune cell subtypes comparing patients with large and small PNH cell fractions by GSEA. The color scale indicates mean NES differences between the two groups, and dot sizes indicate FDR values. Non-significant pathways (FDR ≥ 0.20) are shown in grey. DCs dendritic cells, Mono monocytes, Neut neutrophils, NK natural killer cells, Plasma plasma cells.

We next investigated transcriptomes of enriched BM immune cells by scRNA-seq. After quality control, a total of 190,032 cells were retained for further analyses, comprising 62,694 GPI(−) and 76,593 GPI(+) cells from eight patients, and 50,745 cells from healthy donors (Fig. 4A). Based on scRNA-seq gene expression, cells were assigned to subpopulations of enriched lineage^+^ immune cells: neutrophils, monocytes, CD4^+^ T cells, CD8^+^ T cells, B cells, plasma cells, NK cells, erythroblasts, and dendritic cells (DCs) (Fig. 4A and Supplementary Fig. 6). In differential abundance analysis, GPI(−) cell populations were enriched in neutrophils, monocytes, erythroblasts, NK cells, and DCs, as compared to GPI(+) cell populations (Fig. 4B). By flow cytometry, frequency of GPI(−) neutrophils and monocytes, but not other immune cell subtypes, reflected percentages of GPI(−) HSCs (Fig. 4C and Supplementary Fig. 7). Consistent with previous studies, the percentages of GPI(−) cells were very low in mature lymphoid cells [23, 24], especially CD3^+^ T cells, indicating skewed myeloid lineage differentiation of GPI(−) HSCs. However, genes associated with apoptosis and the cell-cycling pathways were not apparently dysregulated in the limited number of GPI(−) T cells as compared to GPI(+) T cells (Supplementary Fig. 8 and Supplementary Table 5), against positive selection of mt*PIGA* GPI(−) T cells.

GSEA of BM immune cells showed upregulation of the immune response pathways in both GPI(+) and GPI(−) immune cells from PNH patients, especially in lymphoid cells such as CD4^+^ T, CD8^+^ T, and NK cells, as compared to immune cells from healthy donors (Fig. 4D and Supplementary Table 6), suggesting persistent inflammation of BM long after disease onset. When gene expression of immune cells was compared between patients with large and small PNH cell fractions, upregulation of the IFN-γ response pathway was observed in most immune cells from patients with large PNH cell fractions (Fig. 4E and Supplementary Table 7): IFN-γ mediated immune response across immune cell types mainly contributes to selection of targeted BM stem cells.

### Enhanced cell-cell interactions among HSPCs and immune cells

**Fig. 5:**
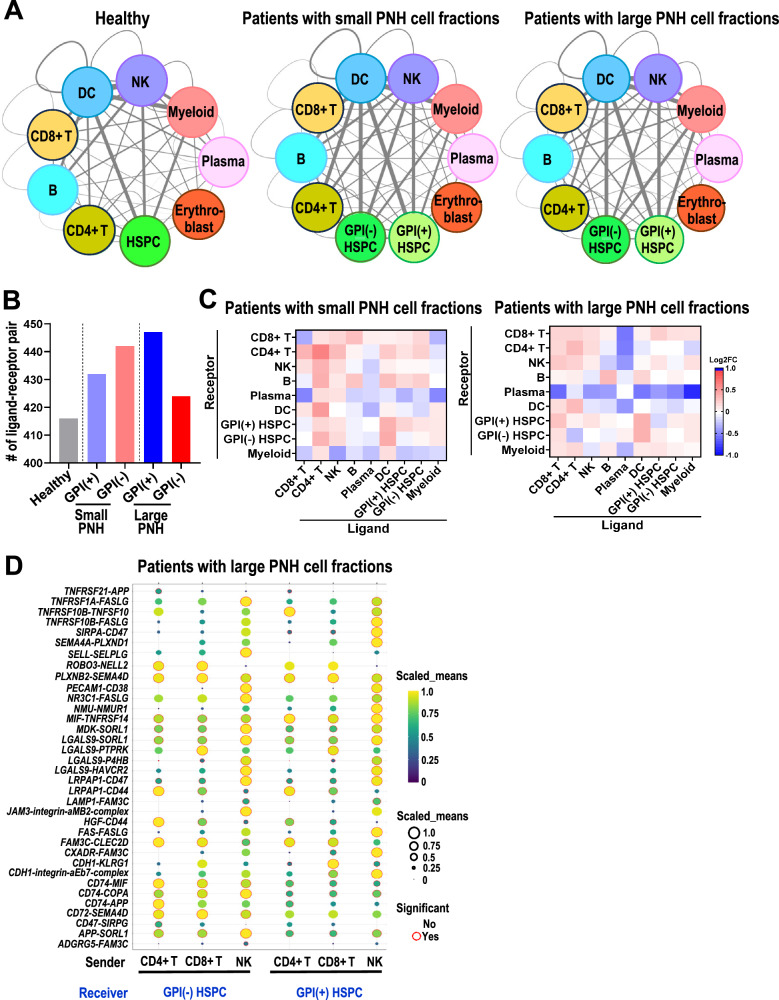
Enhanced cell-cell interactions in PNH patients compared with healthy donors. **A** Network representation of ligand-receptor pairs among bone marrow (BM) cell types estimated by CellPhoneDB. Line thickness indicates the total number of ligand-receptor pairs between two cell types. Neutrophils and monocytes are grouped as myeloid cells. Overall, more ligand-receptor interactions were observed among BM cell types in PNH patients (middle and right) than in those of healthy donors (left). **B** A bar chart showing the number of ligand-receptor pairs between HSPCs and other BM immune cell types. **C** Heatmaps showing log2FC of cell-cell interaction scores inferred using CellPhoneDB across all BM cell types in patients with small and large PNH cell fractions. **D** Ligand-receptor pairs that were overrepresented in PNH patients with large PNH cell fractions compared with healthy controls among CD4^+^ T, CD8^+^ T, NK cells (as sender), and HSPCs (as receiver). Significance indicates whether a ligand-receptor pair is over-represented in patient samples compared with healthy donor samples. Myeloid myeloid cell, Plasma plasma cell.

As IFN-γ has important roles in promoting “cross-talk” among immune cell compartments, we imputed interactions among BM immune cell types and HSPCs with CellPhoneDB [18]. Interactions among cell types were more abundant in PNH patients than in healthy donors (Fig. 5A). In patients with large PNH cell fractions, GPI(+) HSPCs had more significant cell-cell interactions than did GPI(−) HSPCs, but less in patients with small PNH cell fractions (Fig. 5B). Compared to healthy donors, cell-cell interactions were enhanced between GPI(+) HSPCs and effector cells including T cells and NK cells in patients with large PNH cell fractions (Fig. 5C). In contrast, in patients with small PNH cell fractions, enhanced interactions with HSPCs were between GPI(−) HSPCs and effector cells (Fig. 5C).

We plotted over-represented interactions of HSPCs with T cells and NK cells in both patients with large and small PNH cell fractions. In patients with large PNH cell fractions, there were many overrepresented molecular pairs between GPI(+) HSPCs and T cells as well as between GPI(−) HSPCs and T cells (Fig. 5D and Supplementary Fig. 9). Similar interactions were also seen between NK cells and both GPI(+) and GPI(−) HSPCs, indicating both GPI(+) and GPI(−) HSPCs were stressed by these effector cells. Notably, death receptor-mediated apoptosis interactions (e.g., *FAS*–*FASLG* and *TNFRSF10B*–*TNFSF10*) were significantly upregulated between GPI(+) HSPCs and both CD4^+^ T cells and NK cells, suggesting stronger immunological effects on GPI(+) HSPCs than on GPI(−) HSPCs in patients with large PNH cell fractions. In patients with small PNH cell fractions, overrepresented interactions of HSPCs with both T cells and NK cells were very similar to those of patients with large PNH cell fractions, and there were few differences in significant interactions with effector cells between GPI(+) and GPI(−) HSPCs (Supplementary Fig. 10).

### Clonal dynamics of *PIGA* mutated clones

**Fig. 6:**
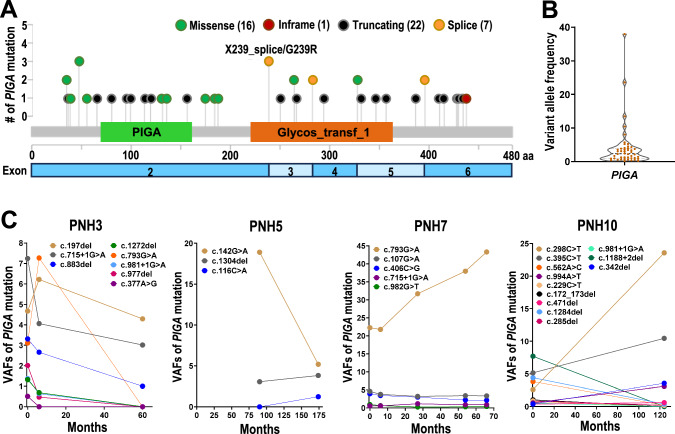
Error-corrected sequencing (ECS)-based detection of *PIGA* mutations. **A** A lollipop plot showing locations of 48 *PIGA* mutations detected in eight PNH patients. **B** A violin plot showing variant allele frequencies (VAFs) of the 48 *PIGA* mutations. **C** Clonal dynamics of *PIGA* mutations in four patients over the follow-up period.

GPI(−) cell populations generally consist of multiple mt*PIGA* clones [25, 26], but these could not be identified in scRNA-seq data due to the extremely low expression of the *PIGA* gene (data not shown). To confirm the presence of *PIGA* mutations in our patients, we performed ECS and identified 48 mutations in the *PIGA* gene in patients’ blood samples; the median number of *PIGA* mutations per patient and their VAFs were 5 (range, 3–10) and 2.31% (range, 0.18–37.9%), respectively (Fig. 6A, B and Supplementary Table 8). There was no difference in the number and the spectrum of *PIGA* mutations between patients with large and small PNH cell fractions, indicating that neither the number nor type of *PIGA* mutations is associated with clonal expansion of mt*PIGA* clones. Five of the eight PNH patients also harbored concomitant somatic mutations in myeloid-cancer genes, mostly *DNMT3A*, but with VAFs less than 5% in most cases (Supplementary Table 8).

In four patients in whom serial samples were available, *PIGA* mutations detected at AA/PNH diagnosis were present over 60-120 months follow-up (Fig. 6C and Supplementary Table 9). As expected, clonal dynamics of mt*PIGA* clones were variable among patients. In PNH 5, whose overall PNH clone size by flow cytometry decreased during follow-up, only a dominant mt*PIGA* clone (*PIGA* c.142G>A) decreased over time, whereas other mt*PIGA* clones slightly increased. In PNH 7, whose overall PNH clone size increased during follow-up, a dominant mt*PIGA* clone (*PIGA* c.793G>A) present at baseline expanded over time and other mt*PIGA* clones were stable. Notably, a very small mt*PIGA* clone (*PIGA* c.982G>T) was repeatedly detected in blood over time with VAFs of 0.2-0.4%.

## Discussion

Although miniscule numbers of PNH cells are present in most normal individuals [27–29], PNH clones have no intrinsic growth advantage in vitro and obviously do not expand in healthy persons in vivo [6–8]. Clonal expansion of mt*PIGA* clones occurs only in the context of immune-mediated BM failure: clones of variable sizes are present in most immune AA patients, and AA/PNH has long been recognized as a distinct syndrome [30–32]. In classical PNH dominated by hemolysis, there may be subclinical marrow failure or a history of AA. Several immunological proof-of-concept scenarios have been proposed to explain PNH clonal expansion: NK cell-mediated cytotoxicity [33], CD1d-restricted, GPI-specific T cells [34], and CD4^+^ T cell-mediated immunologic attack [35]. NK cells also have been implicated in CD8^+^ T cell-mediated autoimmunity in AA patients [36].

Our scRNA-seq data support a model of extrinsic, immune-driven pathophysiology, and specifically immune escape by GPI(−) cells. In patients with large PNH cell fractions, in contrast to patients with small PNH cell fractions, upregulation of the immune response and the apoptosis pathways, and downregulation of the cell-cycling pathways were seen in GPI(+) HSPCs, but not in GPI(−) HSPCs. In addition, BM effector cells, especially CD4^+^ T, CD8^+^ T, and NK cells, were more activated in patients with large PNH cell fractions than in patients with small PNH cell fractions, and they showed enhanced cell-cell communications with GPI(+) HSPCs. Detailed examinations of gene expression profiles for HSCs also supports an extrinsic model and a strong link to autoimmunity: genes associated with the IFN-γ response and the apoptosis pathways were upregulated in GPI(+) HSCs from patients with large PNH cell fractions as compared to those from patients with small PNH cell fractions, but not in GPI(−) HSCs. GPI(−) HSCs appeared relatively similar in their patterns of transcription in patients with large and small GPI(−) cell fractions, but wild-type GPI(+) HSCs showed much more pronounced differences between patients with large and small PNH cell fractions. These transcriptional differences likely reflect secondary associations with clonal expansion, rather than causality. In one case report of a classical hemolytic PNH patient, mt*PIGA* clones present at diagnosis dramatically decreased after syngeneic BM transplantation and did not expand for more than 4 years [37], suggesting relief of immunological stress on GPI(+) HSCs. Similarly, IST has been reported to cause a reduction in PNH clone sizes in patients with AA [38]. These observations are consistent with a concept in clonal evolution that in the absence of reproduction, fitness differences can only emerge if the population undergoes a continuous reduction in size [39]. Strong negative selection by effector immune cells of GPI(+) HSCs may contribute to the relative expansion of mt*PIGA* HSCs.

In our patients with small PNH cell fractions, downregulation of HLA class II genes in wild-type GPI(+) HSCs was observed as compared to GPI(−) HSCs. In a previous study of AA patients, loss of expression of HLA-DR was observed in GPI(+) HSPCs, not in GPI(−) HSPCs [40]. IFN-γ treatment restored the expression of HLA-DR in the affected cells in vitro, suggesting that an epigenetic mechanism might underlie loss of HLA-DR in stem cells. In PNH patients whose mutated clones do not expand, the downregulation of HLA class II genes in GPI(+) HSCs, potentially through an epigenetic mechanism, might contribute to immune evasion by GPI(+) HSCs and alter the balance between GPI(+) and GPI(−) HSCs.

For clonal hematopoiesis in general, both cell-intrinsic and -extrinsic mechanisms have been hypothesized to confer competitive advantages at the level of stem cells [41–43]. Intrinsic mechanisms are favored by analogy with malignancies, as clonal hematopoiesis of indeterminate potential (CHIP) mutations by definition occur in genes known to be mutated in myeloid cancers. With normal aging, clonal expansion might occur due to cells deficient in differentiation potential, apoptosis, or cell-cell regulation [44]. The environment has been implicated in “inflame-aging”, in the putatively healthy elderly and in autoimmune and autoinflammatory diseases [20, 45]. In a single-cell multi-omics study of a large number of CHIP patients, HSPCs from patients with higher VAF CHIP mutations had increased inflammatory signatures as compared to patients with lower VAF CHIP mutations, and immune dysregulation favoring clonal growth was observed particularly in patients with higher VAF CHIP mutations [46]. In PNH, the local environment is autoimmune, dominated but not restricted to cytotoxic T cell targeting of HSCs. Small PNH clones show evidence of immune attack, which large PNH clones appear to have escaped and expanded. Considering both intrinsic and extrinsic mechanisms proposed for clonal hematopoiesis, our data suggest that in PNH clonal expansion is largely shaped by selection pressure from the BM microenvironment. Our recent report of scRNA-seq in AA also demonstrates strong immune-mediated pressure on HSPCs, with IFN-γ–related and apoptosis pathways enriched in the susceptible populations [47]. However, while in AA these signatures are prominent in residual normal HSPCs, in PNH they are more pronounced in GPI(+) HSPCs, allowing GPI(−) clones to expand under immune escape.

Animal models have provided only modest insights into PNH pathophysiology, and by mainly negative findings. In *PIGA* conditional knock-out (CKO) mouse models, mt*PIGA* HSCs reconstitute hematopoiesis without lineage skewing [6, 8]. In addition, a proportion of GPI(−) T cells remains at a relatively high level for life in the *PIGA* CKO heterozygotes mice [8]. Our scRNA-seq data showed potential of GPI(−) HSCs to differentiate in all lineages, including lymphoid lineage, similar to that of GPI(+) HSCs, which was confirmed by flow cytometry analysis. However, the proportion of GPI(−) T cells was very low, despite a high proportion of GPI(−) lymphoid progenitors in marrow, also previously reported [23, 24, 48, 49]. Similarity in gene expression of apoptosis and cell-cycling pathways between GPI(+) and GPI(−) T cells indicates that GPI(−) T cells are not at a low level due to a higher proliferation rate of GPI( + ) T cells than GPI(−) T cells during terminal T cell differentiation. The lack of expansion of GPI(−) T cells likely reflects a mechanism distinct from hematopoietic clones: whereas GPI(−) HSCs are selected through immune escape, T-cell clones may depend more on antigen-specific cell-cell interactions.

From our ECS data, the majority of PNH patients had multiple *PIGA* mutations, as many as 10 in a single patient, strongly implicating selection in the immune environment of marrow failure. Most *PIGA* mutations were not at high VAFs ( > 10%) and did not co-occur with mutations associated to myeloid malignancies at single cell level [50, 51], consistent with other evidence that mt*PIGA* clones infrequently acquire a malignant phenotype: the biology of the GPI(−) cells may be unfavorable for cancer development [52]. Our longitudinal *PIGA* sequencing showed dynamics of mt*PIGA* clones were variable regardless of the types of *PIGA* mutations and concomitant mutations. From a report of whole genome sequencing of single cell-derived colonies of BM samples from two PNH patients, mt*PIGA* clones and even non-mt*PIGA* clones expanded concurrently after AA diagnosis [53]. This finding suggests that immune-mediated selection pressure against normal HSCs, rather than genetic alterations within mt*PIGA* clones, contributes to the expansion of mt*PIGA* clones, although the potential contribution of additional intrinsic factors—such as unidentified genetic or epigenetic alterations—remains unclear and warrants further investigation.

For the current work, among obvious limitations of patient numbers, length of follow-up, and drop out of RNA sequences, we note more specific difficulties. First, the majority of our PNH patients had a history of IST and treatment with complement inhibitors; these therapies might affect transcription in immune cells as well as HSPCs. Nevertheless, scRNA-seq analysis allowed us to directly compare GPI(−) cells with GPI(+) cells within individuals, which were equally subjected to drug and biological exposure. Second, the numbers of both GPI(−) and GPI(+) HSCs were very low in our patients, which limited validation of scRNA-seq data with other methods such as bulk RNA-seq. scRNA-seq analysis of enriched HSCs from additional PNH samples will help to better understand disease pathophysiology of PNH. Third, *PIGA* genotype could not be assigned at the single-cell level due to the very low expression of the *PIGA* gene and other limitations of the platform, we could not determine whether GPI(−) HSCs are transcriptionally homogeneous or different between dominant and non-dominant mt*PIGA* clones within the same patient. Because scRNA-seq mutation-calling requires sufficient transcript coverage, the low expression of *PIGA* makes reliable genotyping technically unfeasible. Future single-cell multi-omics approaches that integrate mutational and transcriptional data will be required to overcome this limitation. We also could not perform longitudinal scRNA-seq analysis in more cases due to the unavailability of fresh serial BM samples from patients. As this study was based on scRNA-seq analysis of BM samples obtained at a single time point for each patient, longitudinal scRNA-seq analysis of BM samples would be informative in evaluating distinct genetic patterns and functional alterations within clones. Finally, no established criteria exist for classification of patients with small versus large PNH cell fractions that correlate perfectly and definitely with clinical features; therefore, we applied an arbitrary cutoff of 50% to BM cells based on prior literatures and the distribution of PNH cell fractions in the current patient cohort. Whether classification should be based on single or serial measurements, and how to account for discrepancies of GPI(−) cell fractions between BM and PB or among different cell populations, remains an open question.

The current work is distinctive in disclosing gene expression variability in hematopoietic cells with a mutated clone size. Our results are the clearest depiction to date of functional changes in hematopoietic clones, expanding in a well described immune and inflammatory environment. Our work provides not only a better understanding of PNH pathophysiology but also clues for studying clonal selection of mutant cells in other clonal hematologic diseases. Clinical and experimental studies, including genetic and immunological approaches, will be important to further elucidate the mechanisms underlying clonal expansion in PNH and in other hematological conditions, both malignant and non-malignant.

## Supplementary information

Supplementary Materials

Supplementary Tables

## Data Availability

Raw and analyzed sequencing data from this study have been deposited in the National Center for Biotechnology Information Gene Expression Omnibus database (accession code GSE248025) and Sequence Read Archive (accession code PRJNA1041418). All other relevant data supporting the key findings of this study are available within the article.
