# Supplementary material for: Imputing causality and clonal dynamics from single-cell transcriptomics in paroxysmal nocturnal hemoglobinuria
Source: Leukemia. 2026 Apr 22;40(7):1368–80. doi: 10.1038/s41375-026-02914-5 (PMC13323074; doi:10.1038/s41375-026-02914-5)
Supplement: Supplementary file 1 — Supplementary Materials [file 41375_2026_2914_MOESM1_ESM.docx]

**SUPPLEMENTARY INFORMATION**

**Imputing causality and clonal dynamics from single-cell transcriptomics in paroxysmal nocturnal hemoglobinuria**

Hiroki Mizumaki^1,*^, Shouguo Gao^1,*^, Zhijie Wu^1^, Fernanda Gutierrez-Rodrigues^1^, Lemlem Alemu^1^, Diego Quinones Raffo^1^, Ivana Darden^1^, Olga Rios^1^, Jennifer Lotter^1^, Sachiko Kajigaya^1^, Jibran Durrani^1,2^, Emma M. Groarke^1^, Bhavisha A. Patel^1^ and Neal S. Young^1^

^1^ Hematology Branch, National Heart, Lung, and Blood Institute, National Institutes of Health, Bethesda, MD, United States

^2^ Division of Hematology, Sylvester Comprehensive Cancer Center, University of Miami Miller School of Medicine, Miami, FL, United States

^*^ These authors contributed equally

**SUPPLEMENTARY METHODS**

**Bone marrow (BM) processing**

BM specimens were obtained from patients and healthy donors, and processed within 6 h after collection. BM mononuclear cells (BMMNCs) were isolated from each person by Ficoll-Hypaque density gradient centrifugation using Ficoll-Paque Premium mononuclear cell separation medium (#17544202, Cytiva, Marlborough, MA, USA). Briefly, BM samples diluted twofold with phosphate-buffered saline (PBS) (#10010031, Thermo Fisher Scientific, Rockville, MD, USA) were layered on top of 1 volume Ficoll-Paque medium in a 50-mL Falcon tube and centrifuged at 1,140 × *g* for 30 min at room temperature with brake-off. A BMMNC layer was isolated and washed with PBS after red blood cell lysis using ACK lysing buffer (#118-156-101, Quality Biological, Gaithersburg, MD, USA). BMMNCs were resuspended in IMDM (#12440-053, Thermo Fisher Scientific) supplemented with 2% fetal bovine serum (FBS) (#12306C, Sigma-Aldrich, Burlington, MA, USA) before fluorescence-activated cell sorting (FACS). Our sorting strategy is illustrated in Supplementary Fig. 2. To enrich GPI(-) and GPI(+) lineage^-^CD34^+^ hematopoietic stem and progenitor cells (HSPCs) with FACS, BMMNCs were stained with the following monoclonal antibodies for 30 min on ice: anti-human lineage cocktail (CD3, CD14, CD16, CD19, CD20, and CD56; clones UCHT1, HCD14, 3G8, HIB19, 2H7, and HCD56, respectively; Cat# 348805, Biolegend, San Diego, CA, USA) in Pacific Blue, anti-human CD64 (clone 10.1; Cat# 983214, Biolegend) in Pacific Blue, anti-CD34 (clone 581; Cat# 555822, BD Biosciences, San Diego, CA, USA) in PE, anti-human CD38 (clone HIT2; Cat# 555462, BD Biosciences) in APC, and anti-human CD59 (clone p282 (H19); Cat#560954, BD Bioscience) in FITC. For sorting of GPI(-) and GPI(+) lineage^+^ immune cells, BMMNCs were stained with the following monoclonal antibodies for 30 min on ice: anti-human lineage cocktail (CD3, CD14, CD16, CD19, CD20, and CD56; clones UCHT1, HCD14, 3G8, HIB19, 2H7, and HCD56, respectively; Cat# 348805, Biolegend) in Pacific Blue, anti-human CD64 (clone 10.1; Cat# 983214, Biolegend) in Pacific Blue, and FLAER (Alexa 488 proaerolysin variant; Cat# FL2S, Cedarlane, Burlington, NC, USA). Cells were sorted using the FACSAria Fusion Flow Cytometer (BD Biosciences) and were subjected to single-cell RNA sequencing (scRNA-seq). Aliquots of BMMNCs were subjected to multi-color flow cytometry to profile HSPCs and BM immune cells.

**Flow cytometry**

BMMNCs were stained with antibody mixtures on ice for 30 min in RPMI 1640 (Cat# 11875093, Thermo Fisher Scientific). Samples were subsequently acquired using the BD LSR Fortessa cytometer (BD Biosciences), followed by post-acquisition analysis using FlowJo software (v.7.6.4; Flowjo LLC, Ashland, OR, USA). Our gating strategy is illustrated in Supplementary Fig. 4B. Antibodies used for flow cytometry profiling of HSPCs were: anti-human lineage cocktail (CD3, CD14, CD16, CD19, CD20, and CD56; clones UCHT1, HCD14, 3G8, HIB19, 2H7, and HCD56, respectively; Cat# 348805, BioLegend) in Pacific Blue, anti-human CD34 in PE (clone 58; Cat# 550761, BD Biosciences), anti-human CD38 in APC (clone HIT2; Cat# 555462, BD Biosciences), anti-human CD10 in BV605 (clone HI10A; Cat# 562978, BD Biosciences), anti-human CD135 in PE/Cy7 (clone BV10A4H2; Cat# 313314, BioLegend), anti-human CD45RA in BV510 (clone HI100; Cat# 304142, BioLegend), and anti-human CD59 (clone p282 (H19); Cat#560954, BD Bioscience) in FITC.

For immune cells, BMMNCs were stained with antibody mixtures on ice for 30 min in RPMI 1640 (Cat# 11875093, Thermo Fisher Scientific). Samples were subsequently acquired using the BD LSR Fortessa cytometer (BD Biosciences), followed by post-acquisition analysis using the FlowJo software (Supplementary Fig. 7). Antibodies used were: anti-human CD45 in APC (clone HI30; Cat# 555485, BD Biosciences), anti-human CD11b in APC-Cy7 (clone ICRF44; Cat# 301342, BioLegend), anti-human CD33 in BV510 (clone WM53; Cat# 303422, BioLegend), anti-human CD56 in PE/Cy7 (clone 5.1H11; Cat# 362510, BioLegend), anti-human CD3 in Pacific Blue (clone HIT3a; Cat# 300330, BioLegend), anti-human CD19 in PE (clone H1B19; Cat# 302208, BioLegend), FLAER (Alexa 488 proaerolysin variant) (Cat# FL2S, Cedarlane), and 7-AAD (Cat# 00-6993-50, Thermo Fisher Scientific).

**Preparation and sequencing of scRNA-seq library**

FACS sorted cells were subjected to scRNA-seq analysis using the Chromium Next GEM Single Cell 3' Reagent Kits v3.1 (Single Index) System (10x Genomics, Pleasanton, CA, USA), following the manufacturer’s protocols. In brief, sorted cells were washed with 1× PBS supplemented with 0.04% FBS. Cell concentrations and viabilities were determined using the trypan blue staining method and the LUNA-II™ Automated Cell Counter (Logos Biosystems, Annandale, VA, USA). Cells were loaded onto a Chromium Chip G and subjected to single-cell partitioning, lysis, and barcoding using the Chromium^TM^ Single Cell Controller (10x Genomics). Subsequently, cDNA was generated in a thermal cycler and purified with Dynabeads MyOne SILANE, followed by cDNA amplification. cDNA was allocated for gene expression library construction (10 μL of cDNA). Any remaining cDNA was stored in a freezer. For gene expression library construction, amplified cDNA was fragmented, end-repaired, and A-tailing double-sided size-selected with solid phase reversible immobilization (SPRI)-select beads. Qualities and quantities of the libraries were assessed using the Agilent 2100 Bioanalyzer (Agilent Technologies, Santa Clara, CA, USA). Gene expression libraries were pooled to achive approximately 20,000 reads per cell, and sequenced using the NovaSeq 6000 system (Illumina, San Diego, CA, USA) with read lengths of 28-bp (read 1), 8-bp (i7 index), 0-bp (i5 index), and 91-bp (read 2).

**scRNA-seq data preprocessing and quality control**

After single-cell libraries were sequenced using the Illumina system, the Cellranger ver 7.0.1 pipeline (https://www.10xgenomics.com/support/software/cell-ranger/latest) was utilized to process scRNA-seq raw data for read alignment to the genome and generation of gene-cell expression matrices^1^. Specifically, sequencing reads were aligned to the human reference genome (hg38) using the STAR aligner^2^ with annotation by Ensembl. The uniquely aligned reads were used to measure expression levels for all ENSEMBL genes. Low-quality cells were excluded from further analyses if the number of genes detected was < 500 genes/cell (potential fragments), > 6,000 genes/cell (potential doublets), or > 5% mitochondrial reads/cell, and the remaining cells were subjected to subsequent data analysis. Downstream analyses were mainly performed using the R software package^3^ in Seurat^4^ (<http://satijalab.org/seurat/>, v4.0.4) on lineage^-^CD34^+^ cells and lineage^+^ cells, separately. Raw reads in individual cells were first scaled by a library size to 10,000 and then log-transformed. To improve downstream dimensionality reduction and clustering, we performed data integration with canonical correlation analysis (CCA) in the Seurat package with functions of FindIntegrationAnchors and IntegrateData to remove a batch effect. For Principal Component Analysis (PCA) of high-dimensional data, 2000 highly variable genes were used. Top 50 principal components were selected for unsupervised clustering of cells with a graph-based clustering approach, followed by further dimensional reduction with UMAP.

**Quantification and statistics**

After alignment with CCA, cells from different subjects were well mixed and separated by cell type categories. Clusters were identified by the FindClusters function in Seurat^4^ and shown in UMAP plots. Accordingly, specific genes in each cluster were identified using the Wilcoxon Rank Sum test implemented in the Seurat package (v.4.0.4).

***Cell type assignment.*** For lineage^-^CD34^+^ cells, a HSPC type was assigned to each cluster based on the significance of overlap between HSPC populations and cluster-specific genes (Fisher’s exact test)^5, 6^. More specifically, top 250 overexpressed genes in each HSPC population were downloaded from http://www.jdstemcellresearch.ca/node/32 as cell-type specific signature genes. Subsequently, the one-tailed Fisher’s exact test was utilized to assess enrichment of HSPC signature genes in the cluster marker gene list for each cluster^7^, and a top associated cell type was assigned to each cluster. Cell types of BM cells were assigned with the same strategies using the Human Cell Atlas as reference^8^. Cell type annotations were manually refined based on known marker genes.

***Differential abundance analysis.*** Differences in cell abundances between the samples of PNH patients and those of the healthy donors were assessed and visualized with the miloR package (https://bioconductor.org/packages/release/bioc/html/miloR.html)^9^. Specifically, the Seurat objects were converted to SingleCellExperiment objects, and the PCAs and UMAPs of Seurat objects were also assigned to the singleCellExperiment objects. Each neighborhood was assigned to a cell-type label based on the majority vote of cells belonging to that neighborhood. A neighborhood was labeled as “Mixed” if the most abundant label was present in < 75% of cells within that neighborhood^9^. We modified the source code of miloR for plotting the results.

***Analysis of sources of transcriptomic variation.*** Gene expression is impacted by a number of biological factors. Decomposing gene expression into percentages attributable to multiple biological sources of variation helps to understand their contribution magnitudes. An expression variance for each gene was partitioned into components attributes to each variable using a linear mixed model implemented in the variancePartition package in Bioconductor^10^. In this study categorical variables (disease status, cell phenotypes, and PNH cell fraction sizes) were modeled as the contributors, and the results were visualized using the ggtern package, an extension of ggplot2.

***Multidimensional scaling (MDS) plot.*** For each sample, we summed gene counts of all its cells to create pseudo-bulk RNA-seq data. Then the plotMDS function in the edgeR package (<https://bioconductor.org/packages/release/bioc/html/edgeR.html>) was used to create a MDS plot^11^, and the squared Euclidean distance in the MDS plot is used to score the dissimilarity among samples.

***Gene Set Enrichment Analysis (GSEA).*** Preranked gene-set analysis on MsigDB v2023.2 Hallmark gene sets was performed using preranked gene lists with GSEA software (<http://software.broadinstitute.org/gsea>). Genes were preranked according to log2 fold change values.

***Reconstruction of hematopoiesis trajectories using scRNA-seq data and dynamic gene expression.*** We used Monocle 2^12^, an established trajectory-detection algorithm, for pseudotime ordering. Monocle 2 orders cells along pseudotime that recapitulates known marker trends along the differentiation. Tracking gene expression, pathway activity, or cell type composition changes along pseudotime enable determination of the differentiation change for each of the terminal fates.

***Projection of patients’ cells to the map of normal hematopoiesis.*** To characterize early hematopoiesis in PNH patents, individual cells were projected onto the map of normal hematopoietic differentiation based on cell-by-cell comparison of patterns of global gene expression and localization to the most similar healthy donor cells with functions of transfer_cell_labels() and fix_missing_cell_labels() in Monocle 2. This strategy was used for mapping patients’ cells on UMAP plots, and assigning pseudotime estimation for cells in individual patients.

***Definition of pathway activity scores.*** We downloaded gene lists of HALLMARK_APOPTOSIS, HALLMARK_G2M_CHECKPOINT, HALLMARK_INTERFERON_GAMMA_RESPONSE, and HALLMARK_INTERFERON_GAMMA_RESPONSE from MSigDB of GSEA. Their activity scores (expression levels) in different samples or cell populations were calculated with the AddModuleScore() function built in the Seurat (<http://satijalab.org/seurat/>). The activity scores were normalized with healthy donors included in individual studies, and the double-sided t-test was used to assess the difference between samples or cell populations.

We also calculated HLA class II genes, the UPR-IRE1 pathway, the PERK pathway, the ATF6 pathway, and HSC activated signature scores based on the following gene sets. The HLA class II genes consist of *HLA-DRA*, *HLA-DRB1*, *HLA-DRB5*, *HLA-DQA1*, *HLA-DQA2*, *HLA-DQB1*, *HLA-DMA*, *HLA-DMB*, *HLA-DPA1,* and *HLA-DPB1*. The UPR-IRE1 pathway genes consist of *HSPA5, ERN1, XBP1, ADD1, YIF1A, TLN1, TPP1, SRPRA, DNAJB11, SYVN1, EXTL3, PREB, EXTL1, TATDN2, DCTN1, DNAJC3, CXXC1, GFPT1, GOSR2, EDEM1, SSR1, C19orf10, SERP1, PPP2R5B, WIPI1, SULT1A3, WFS1, DDX11, GSK3A, HDGF, KLHDC3, FKBP14, SRPRB, DNAJB9, ATPV0D1, TSPYL2, ZBTB17, PDIA5, CUL7, KDELR3, SEC31A, ACADVL, SHC1, PLA2G4B, EXTL2, LMNA, ARFGAP1, HYOU1, CTDSP2,* and *PDIA6*. The PERK pathway genes consist of *HSPA5, EIF2AK3, EIF2S2, EIF2S3, EIF2S1, CXCL8, ATF4, EXOSC3, EXOSC1, EXOSC9, EXOSC6, EXOSC5, EXOSC7, EXOSC4, EXOSC8, DIS3, EXOSC2, KHSRP, DCP2, PARN, ASNS, CEBPG, CEBPB, IGFBP1, DDIT3, NFYA, NFYB, NFYC, ATF6, CCL2, HERPUD1, ATF3,* and *IL8*. The ATF6 pathway genes consist of *MBTPS1*, *MBTPS2*, *HSPA5, ATF4*, *DDIT3*, *NFYA*, *NFYB*, *NFYC*, *ATF6*, *XBP1*, *HSP90B1*, *CALR*, *DDIT3,* and *HSP90B1*. The activated HSC signature gene scores were defined based on the published reference gene set^13^. The module scores were calculated by AddModuleScore() function built in the Seurat^4^.

***Ligand receptor analysis.*** CellPhoneDB (v5.0.0)^14^ was used to identify putative cell–cell interactions of PNH patients and healthy donors, and between different cell populations. The code of Ktplots (<https://github.com/zktuong/ktplots>) was modified to visualize ligand-receptor interacting strength across cell populations from CellPhoneDB results.

**References**

1. Zheng GX, Terry JM, Belgrader P, Ryvkin P, Bent ZW, Wilson R*, et al.* Massively parallel digital transcriptional profiling of single cells. *Nat Commun* 2017; **8:** 14049.

2. Dobin A, Davis CA, Schlesinger F, Drenkow J, Zaleski C, Jha S*, et al.* STAR: ultrafast universal RNA-seq aligner. *Bioinformatics* 2013; **29**(1)**:** 15-21.

3. Team RC. R: A Language and Enviroment for Statistical Computing. R Foundation for Statistical Computing; 2023.

4. Stuart T, Butler A, Hoffman P, Hafemeister C, Papalexi E, Mauck WM, 3rd*, et al.* Comprehensive Integration of Single-Cell Data. *Cell* 2019; **177**(7)**:** 1888-1902 e1821.

5. Laurenti E, Doulatov S, Zandi S, Plumb I, Chen J, April C*, et al.* The transcriptional architecture of early human hematopoiesis identifies multilevel control of lymphoid commitment. *Nat Immunol* 2013; **14**(7)**:** 756-763.

6. Zhao X, Gao S, Wu Z, Kajigaya S, Feng X, Liu Q*, et al.* Single-cell RNA-seq reveals a distinct transcriptome signature of aneuploid hematopoietic cells. *Blood* 2017; **130**(25)**:** 2762-2773.

7. Guo M, Wang H, Potter SS, Whitsett JA, Xu Y. SINCERA: A Pipeline for Single-Cell RNA-Seq Profiling Analysis. *PLoS Comput Biol* 2015; **11**(11)**:** e1004575.

8. Hay SB, Ferchen K, Chetal K, Grimes HL, Salomonis N. The Human Cell Atlas bone marrow single-cell interactive web portal. *Exp Hematol* 2018; **68:** 51-61.

9. Dann E, Henderson NC, Teichmann SA, Morgan MD, Marioni JC. Differential abundance testing on single-cell data using k-nearest neighbor graphs. *Nat Biotechnol* 2022; **40**(2)**:** 245-253.

10. Hoffman GE, Schadt EE. variancePartition: interpreting drivers of variation in complex gene expression studies. *BMC Bioinformatics* 2016; **17**(1)**:** 483.

11. Chen Y, Chen L, Lun ATL, Baldoni P, Smyth GK. edgeR v4: powerful differential analysis of sequencing data with expanded functionality and improved support for small counts and larger datasets. *Nucleic Acids Res* 2025;**53**(2): gkaf018.

12. Qiu X, Mao Q, Tang Y, Wang L, Chawla R, Pliner HA*, et al.* Reversed graph embedding resolves complex single-cell trajectories. *Nat Methods* 2017 Oct;**14**(10):979-982.

13. Garcia-Prat L, Kaufmann KB, Schneiter F, Voisin V, Murison A, Chen J*, et al.* TFEB-mediated endolysosomal activity controls human hematopoietic stem cell fate. *Cell Stem Cell* 2021; **28**(10)**:** 1838-1850 e1810.

14. Garcia-Alonso L, Lorenzi V, Mazzeo CI, Alves-Lopes JP, Roberts K, Sancho-Serra C*, et al.* Single-cell roadmap of human gonadal development. *Nature* 2022; **607**(7919)**:** 540-547.

**
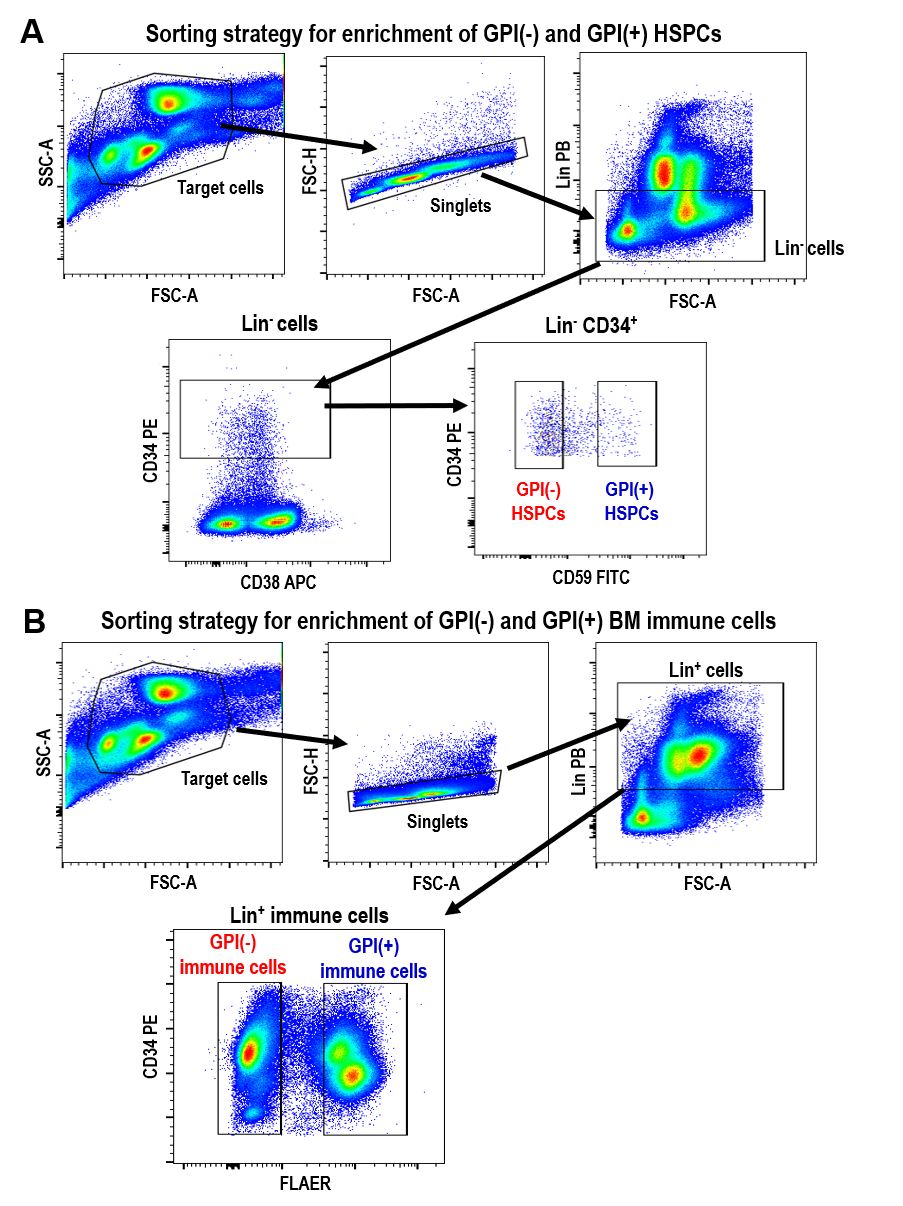
****Supplementary Fig. 1 Gating strategies for flow cytometric cell sorting. A** Representative flow cytometry plots showing a gating strategy for flow cytometric sorting of GPI(-) and GPI(+) hematopoietic stem and progenitor cells (HSPCs). Target cells were gated sequentially as follows: 1. high FSC-A and high SSC-A cells excluding debris; 2. a singlet cell fraction excluding doublet cells; 3. a lineage (Lin)^-^ cell fraction; 4. CD34^+^ HSPCs; and 5. Lin^-^CD34^+^CD59^-^ HSPCs (GPI(-) HSPCs) and Lin^-^CD34^+^CD59^+^ HSPCs (GPI(+) HSPCs). **B** Representative flow plots showing a gating strategy for flow cytometric sorting of GPI(-) and GPI(+) bone marrow (BM) immune cells. Target cells were gated sequentially as follows: 1. high FSC-A and high SSC-A cells excluding debris; 2. a singlet cell fraction excluding doublet cells; 3. Lin^+^ cell fraction; 4. Lin^+^CD59^-^ cells (GPI(-) BM immune cells) and Lin^+^CD59^+^ immune cells (GPI(+) BM immune cells).

**
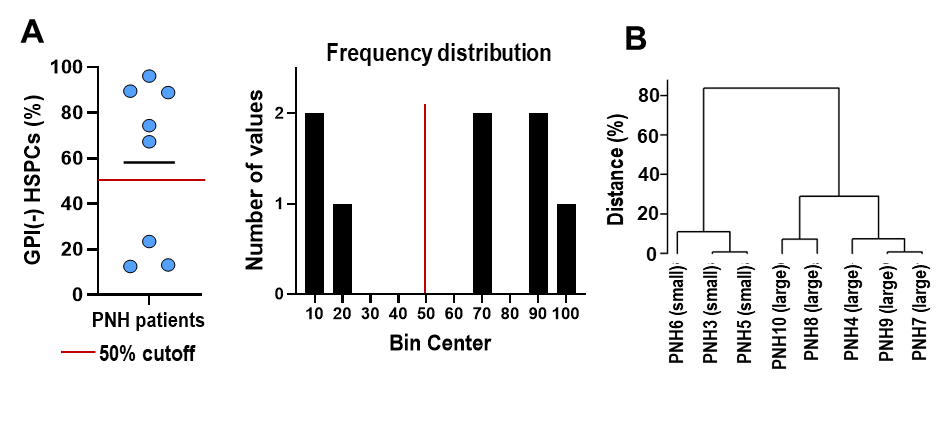
**

**Supplementary Fig. 2 Distribution of percentage of GPI(-) HSPCs in this study.** **A** A dot plot and a frequency distribution plot of GPI(-) HSPC% in the 8 PNH patients. Red line indicates a 50% cutoff. **B** A hierarchical clustering tree of GPI(-) HSPC% in the 8 patients.

**
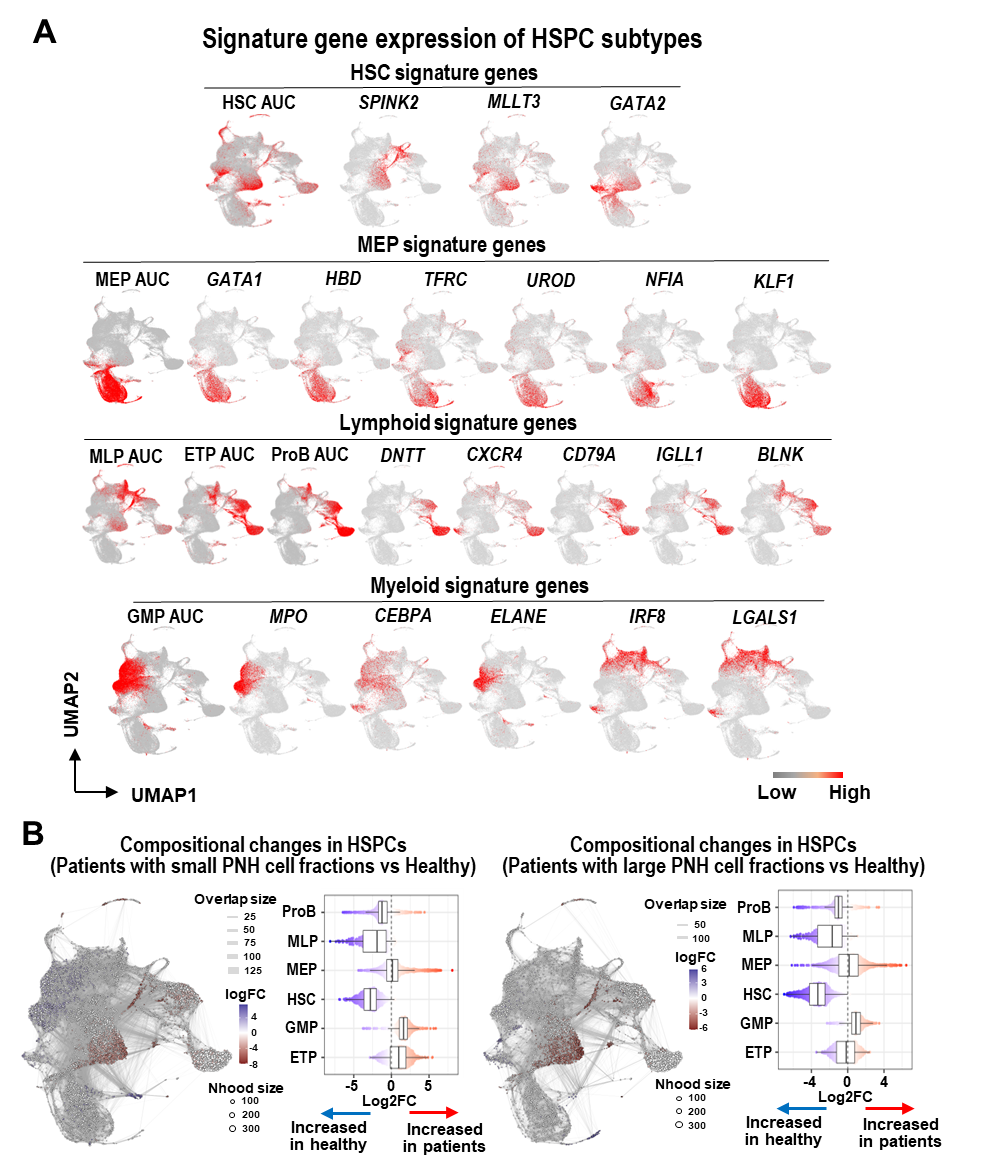
Supplementary Fig. 3 A gene expression profile of HSPC subtypes. A** Signature gene expression of HSPC subtypes. Expression of cell-type specific genes is highlighted in uniform manifold approximation and projection (UMAP) plots of enriched lineage^-^CD34^+^ HSPCs from all subjects. **B** Neighborhood graphs of HSPCs for patients with small PNH cell fractions vs healthy donors (left), and patients with large PNH cell fractions vs healthy donors (right) using the Milo differential abundance testing. Nodes represent neighborhoods from the HSPC population. A color scale indicates log2-fold change (log2FC) differences between PNH patients and healthy donors. Significant changes are colored in blue and red. Nondifferential abundance Nhoods (false discovery rate [FDR] ≥ 0.10) are in white. Beeswarm and box plots of HSPCs based on the Milo differential abundance testing are shown. Analysis was performed similarly to Fig. 2B. A box plot was created in a similar fashion as in Fig. 2B. ETP, early T lineage progenitor; GMP, granulocyte-monocytic progenitor; HSC, stem cells and multipotent progenitors; ProB, B lymphocyte progenitors; MEP, megakaryocyte-erythrocyte progenitor; MLP, multi-lymphoid progenitor.


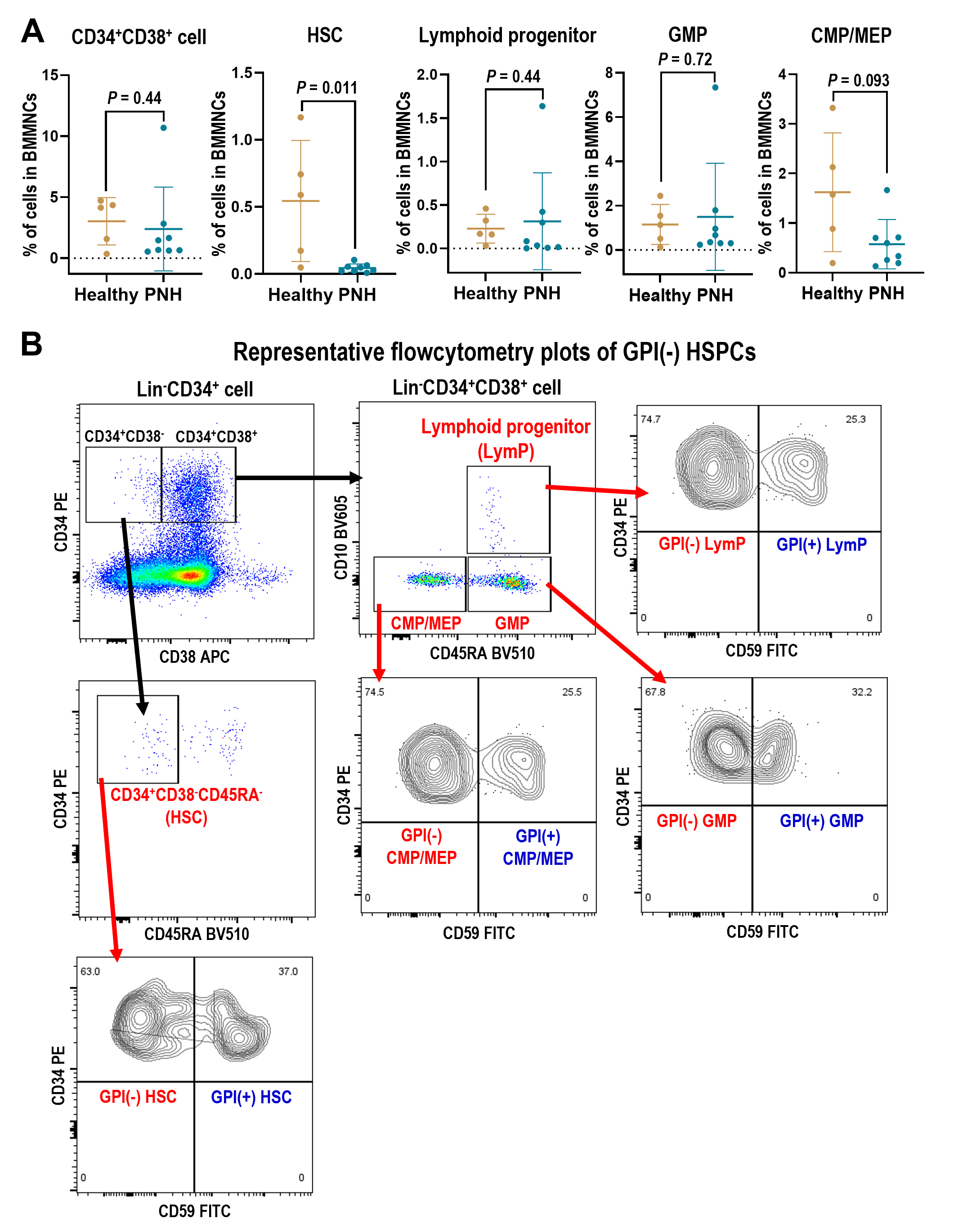
**Supplementary Fig. 4 Differences in cellular compositions in HSPC subtypes between PNH patients and healthy donors. A** Percentages of individual HSPC subtypes relative to a total number of bone marrow mononuclear cells (BMMNCs) derived from PNH patients (*n* = 8, light brown dots) and healthy donors (*n* = 5, blue dots). Data are presented as a mean ± a standard deviation (SD). *P* values were calculated using the two-sided unpaired Mann-Whitney *U* test. **B** Phenotypes of HSPCs in PNH patients by flow cytometry. Cell populations were defined as follows: HSC, lineage (Lin)^-^CD34^+^CD38^-^CD45RA^-^; CMP/MEP, Lin^-^CD34^+^CD38^+^CD10^-^CD45RA^-^; GMP, Lin^-^CD34^+^CD38^+^CD10^-^CD45RA^+^; and lymphoid progenitors, Lin^-^CD34^+^CD38^+^CD10^+^. A GPI(-) HSPC population was identified as a FLAER^-^ cell population in each HSPC subtype. CMP, common myeloid progenitor; GMP, granulocyte-monocytic progenitor; HSC, stem cells and multipotent progenitors; LymP, lymphoid progenitor; MEP, megakaryocyte-erythrocyte progenitor.

**
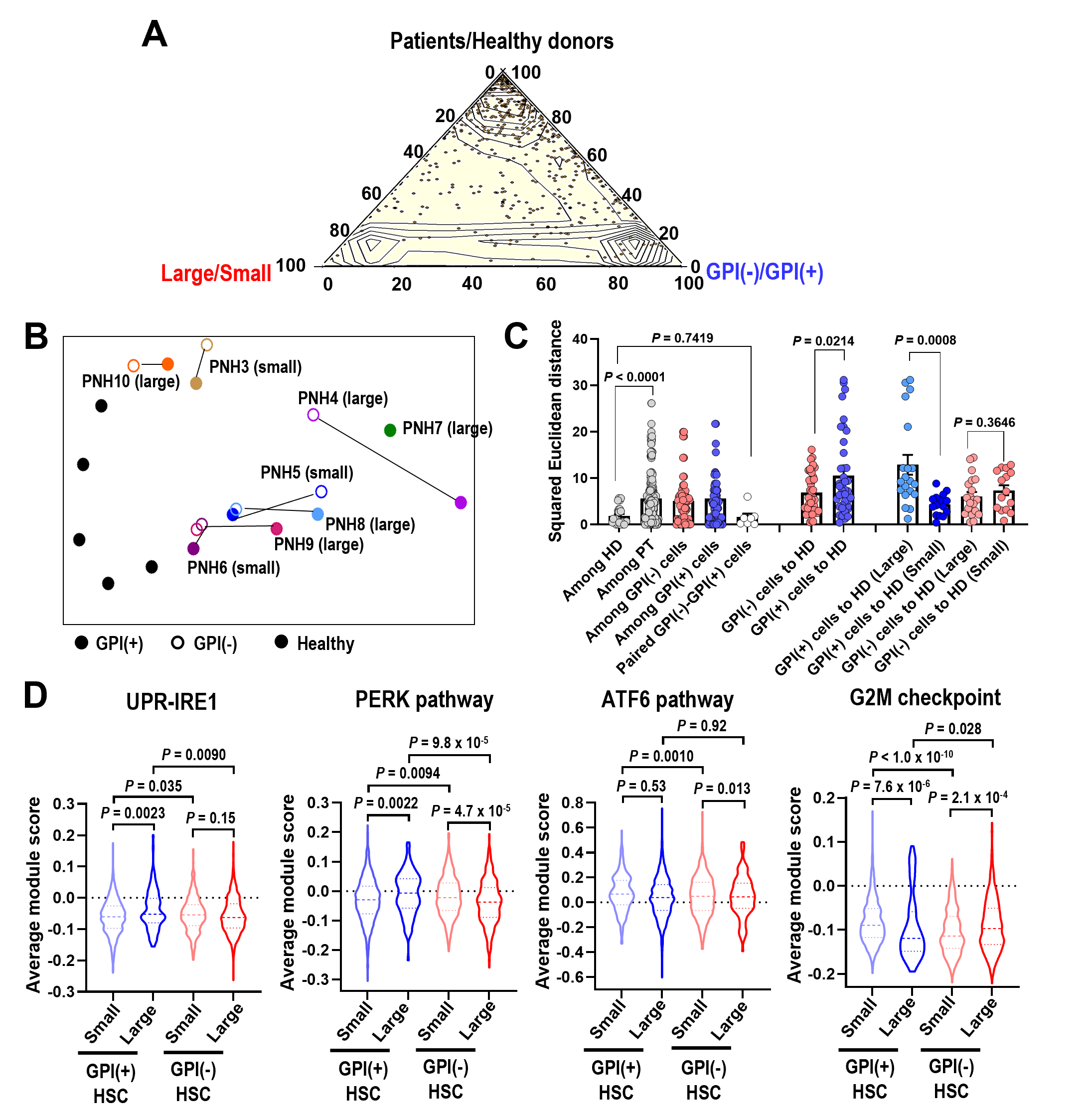
Supplementary Fig. 5 Dysregulated gene profiles in HSPCs from PNH patients. A** A plot showing fractions of variances (gene expression of HSPCs) explained by disease status, cell phenotypes, and GPI(-) HSPC clone size (large vs. small). Each dot represents a gene, and its position reflects how much of its expression variance is explained by disease status (patients vs. healthy donors), cell phenotype (GPI(-) vs. GPI(+) HSPCs), and clone sizes of GPI(-) HSPCs (large vs. small). Contour lines indicate areas where many genes share similar variance-explained patterns. Denser (closely spaced) contours mark regions with a high density of genes that share similar variance patterns (typically reflecting stronger between-group differences for the relevant factor[s]), whereas widely spaced contours indicate sparser regions (generally corresponding to smaller between-group differences for most genes in that area). **B** A multidimensional scaling plot showing scattering and clustering of GPI(+) and GPI(-) cells from patients, and samples from healthy donors, based on whole transcriptome data of HSPCs. Solid dots indicate GPI(+) cells, circles indicate GPI(-) cells; black dots indicate samples from healthy donors; paired GPI(+) and GPI(-) cells from the same patient were connected by solid line; different colors indicate different patients and color legend is the same as in Figure 1C. **C** Squared Euclidean distances on a MDS plot for individual samples by different groups. *P* values were calculated using the two-sided unpaired Mann-Whitney *U* test. **D** Gene set module scores of the unfolded protein response (UPR) pathways (UPR-IRE1, PERK, and ATF6 pathways) and the G2M pathway in HSCs for GPI(+) HSCs in patients with small and large PNH cell fractions, and GPI(-) HSCs in patients with small and large PNH cell fractions. Data are presented as a mean ± SD. *P* values were calculated using the two-sided unpaired Mann-Whitney *U* test. HD, healthy donor; HSC, hematopoietic stem cell; PT, patients.


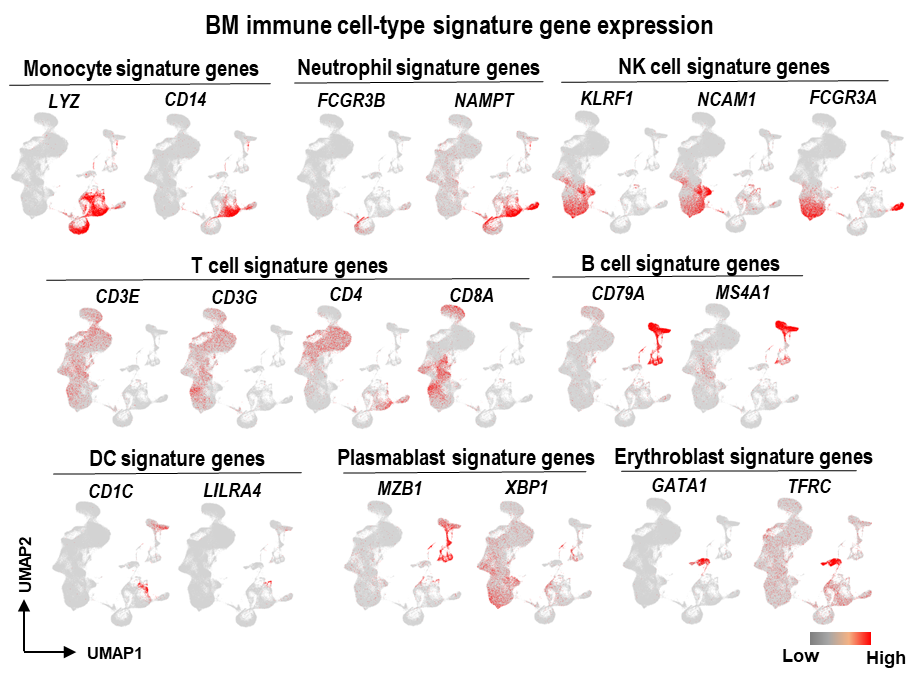


**Supplementary Fig. 6 Gene expression profiles of BM immune cells.** Expression of immune cell-type specific genes is highlighted in UMAP plots of enriched lineage^+^ cells from all PNH patients and healthy donors.

**
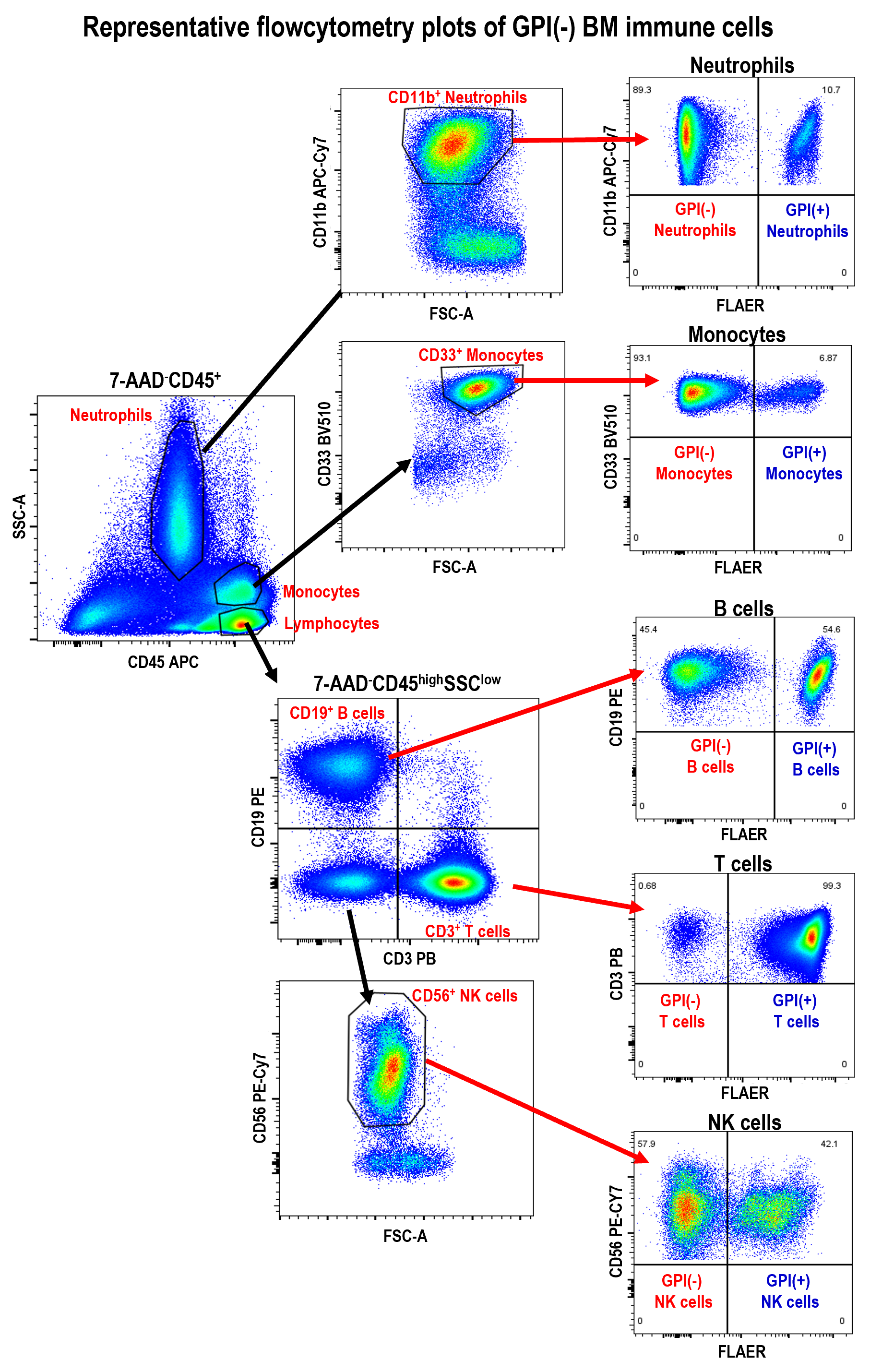
Supplementary Fig. 7 Identification of GPI(-) cell populations in individual BM immune cell subtypes.** Cell populations were defined as follows: neutrophils, CD11^+^ cells; monocytes, CD33^+^ cells; B cells, CD3^-^CD19^+^ cells; T cells, CD3^+^CD19^-^ cells; NK cells, and CD3^-^CD19^-^CD56^+^ cells. A GPI(-) population was identified as FLAER^-^ cells in each cell subtype.

**
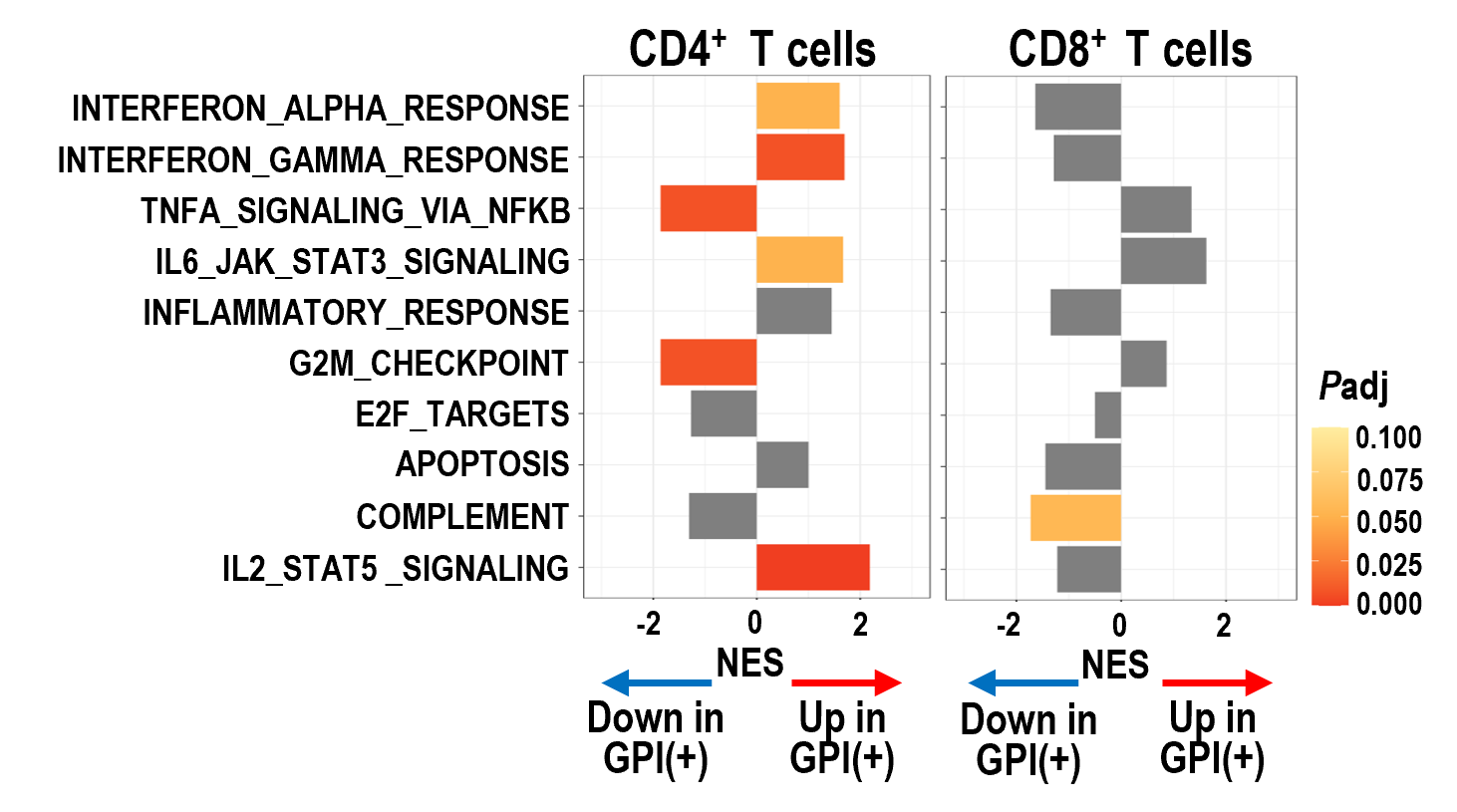
Supplementary Fig. 8 A bar chart of gene set enrichment scores comparing GPI(+) vs. GPI(-) cell populations in CD4^+^ T and CD8^+^ T cell subtypes.** A color scale indicates adjusted *P* values (*P*adj). Non-significant pathways (*P*adj > 0.10) are shown in grey. NES, normalized enrichment score.

**
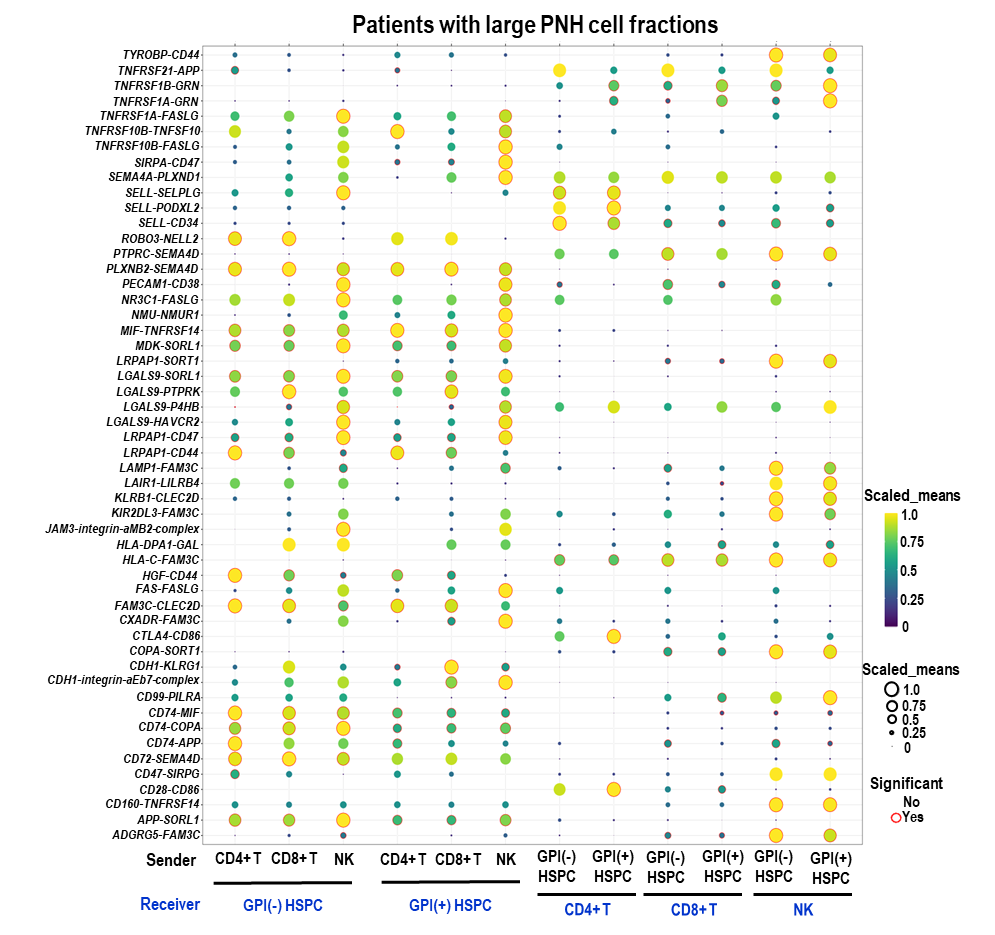
Supplementary Fig. 9 Ligand-receptor pairs that were overrepresented in PNH patients with large PNH cell fractions compared with healthy donors among CD4^+^ T, CD8^+^ T, NK cells, and HSPCs.** Significance indicates if the ligand-receptor pair is over-represented in patient samples compared with healthy donor samples.


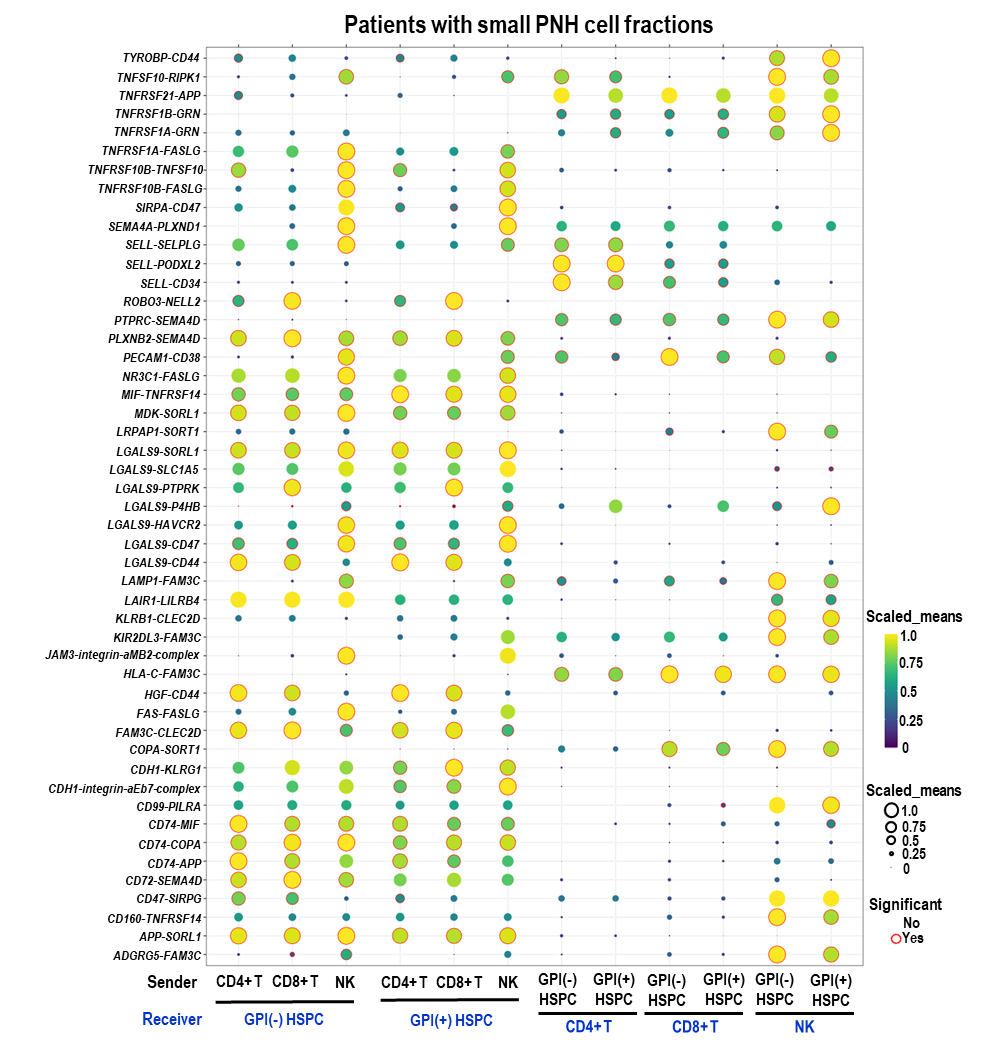


**Supplementary Fig. 10 Ligand-receptor pairs that were overrepresented in PNH patients with small PNH cell fractions compared with healthy donors among CD4^+^ T, CD8^+^ T, NK cells, and HSPCs.** Significance indicates if the ligand-receptor pair is over-represented in patient samples compared with healthy donor samples.
